# Nanosized *T*_1_ MRI Contrast Agent Based on a Polyamidoamine as Multidentate Gd Ligand

**DOI:** 10.3390/molecules27010174

**Published:** 2021-12-28

**Authors:** Paolo Arosio, Davide Cicolari, Amedea Manfredi, Francesco Orsini, Alessandro Lascialfari, Elisabetta Ranucci, Paolo Ferruti, Daniela Maggioni

**Affiliations:** 1 Dipartimento di Fisica, INFN, Istituto Nazionale di Fisica Nucleare-Milano Unit, Università degli Studi di Milano, Via Celoria 16, 20133 Milano, Italy; francesco.orsini@unimi.it; 2 Consorzio Interuniversitario Nazionale per la Scienza e Tecnologia dei Materiali, Via Golgi 19, 20133 Milano, Italy; 3 Dipartimento di Fisica, INFN, Istituto Nazionale di Fisica Nucleare-Milano Unit, Università degli Studi di Pavia, Via Bassi 6, 27100 Pavia, Italy; davide.cicolari01@universitadipavia.it (D.C.); alessandro.lascialfari@unipv.it (A.L.); 4 Dipartimento di Chimica, Università degli Studi di Milano, Via Golgi 19, 20133 Milano, Italy; amedea.manfredi@unimi.it (A.M.); elisabetta.ranucci@unimi.it (E.R.); paolo.ferruti@unimi.it (P.F.)

**Keywords:** polyamidoamine, MRI, relaxivities, Gd-based contrast agent, nanosized contrast agent

## Abstract

A linear polyamidoamine (PAA) named BAC-EDDS, containing metal chelating repeat units composed of two *tert*-amines and four carboxylic groups, has been prepared by the aza-Michael polyaddition of ethylendiaminodisuccinic (EDDS) with 2,2-bis(acrylamido)acetic acid (BAC). It was characterized by size exclusion chromatography (SEC), FTIR, UV–Vis and NMR spectroscopies. The *pK_a_* values of the ionizable groups of the repeat unit were estimated by potentiometric titration, using a purposely synthesized molecular ligand (Agly-EDDS) mimicking the structure of the BAC-EDDS repeat unit. Dynamic light scattering (DLS) and ζ-potential analyses revealed the propensity of BAC-EDDS to form stable nanoaggregates with a diameter of approximately 150 nm at pH 5 and a net negative charge at physiological pH, in line with an isoelectric point <2. BAC-EDDS stably chelated Gd (III) ions with a molar ratio of 0.5:1 Gd (III)/repeat unit. The stability constant of the molecular model Gd-Agly-EDDS (log K = 17.43) was determined as well, by simulating the potentiometric titration through the use of Hyperquad software. In order to comprehend the efficiency of Gd-BAC-EDDS in contrasting magnetic resonance images, the nuclear longitudinal (*r*_1_) and transverse (*r*_2_) relaxivities as a function of the externally applied static magnetic field were investigated and compared to the ones of commercial contrast agents. Furthermore, a model derived from the Solomon–Bloembergen–Morgan theory for the field dependence of the NMR relaxivity curves was applied and allowed us to evaluate the rotational correlation time of the complex (*τ* = 0.66 ns). This relatively high value is due to the dimensions of Gd-BAC-EDDS, and the associated rotational motion causes a peak in the longitudinal relaxivity at ca. 75 MHz, which is close to the frequencies used in clinics. The good performances of Gd-BAC-EDDS as a contrast agent were also confirmed through in vitro magnetic resonance imaging experiments with a 0.2 T magnetic field.

## 1. Introduction

Magnetic resonance imaging (MRI) is an in-clinic diagnostic modality that has received tremendous development for many decades. It allows for obtaining images of an organism in a non-invasive way, without damaging ionizing radiation and with an excellent penetration depth, having the advantage of providing better spatial resolution than other clinical imaging modalities [1,2,3,4,5,6,7,8,9]. Unfortunately, in many cases, the natural tissue contrast is not enough to obtain images of good quality. For that reason, many different contrast agents have been developed since the 1970s. To date, the contrast agents used clinically are small molecules, mostly gadolinium chelates, which, however, have the drawback of a short blood clearance time, which does not allow for assessing the tissue biodistribution. Conversely, binding lanthanide chelates to macromolecules [10,11,12] or nanoparticles [13] can extend their blood permanence, thus improving the ability to passively reach the target. Furthermore, binding many lanthanide chelates to a single macromolecule or nanoparticle improves the image quality as it increases the local concentration of the contrast agent. At the same time, by tailoring the dimension of the nano-construct it is possible to obtain a tumbling frequency of the same order of nuclear Larmor frequency, thus provoking shortening of nuclear relaxation times and consequently an enhancement of the image contrast. Both these two factors contribute to enhancing relaxivity [14]. Likewise, even if the MRI contrast enhancement by means of Gd-based contrast agents (CAs) is nowadays extensively used (in 2018 ≈ 30 million of doses were administered [15]), starting from the second decade of the 2000s their use has been associated with the development of nephrogenic systemic fibrosis in patients with impaired renal function [16], and with the accumulation of Gd in patients exposed recurrently to CAs [17]. Therefore, several researchers are constantly looking for possible alternatives to the current CAs.

PAAs are a family of synthetic polymers obtained by the aza-Michael-type polyaddition of *prim*- or bis-*sec*-amines to bis(acrylamide)s [18]. They are water soluble, biocompatible and biodegradable. Moreover, they show stealth-like behavior [19,20] and many of them self-assemble in water solution giving small nanoaggregates, which have the chance to be passively accumulated at the tumor site by the so-called enhanced permeability and retention effect (EPR) [21]. All these favorable features make PAAs good candidates as effective drug delivery or contrast agent vectors.

**Scheme 1:**
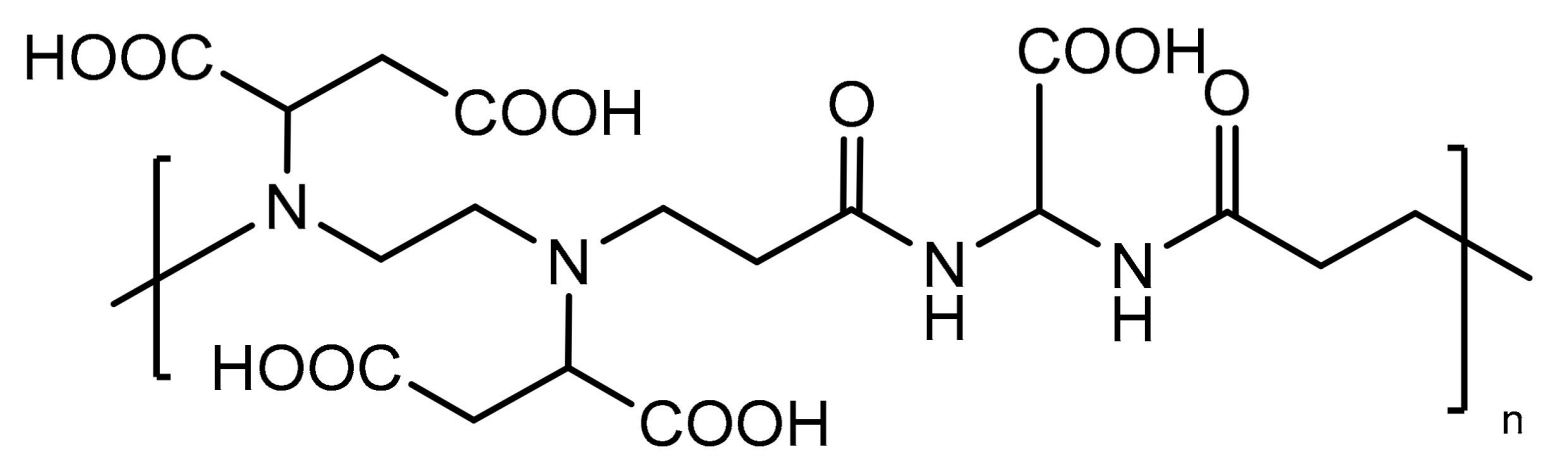
Structure of the repeating unit of BAC-EDDS.

In this work, we synthesized a Gd(III) polymer complex with a multifunctional polyamidoamine (PAA) named BAC-EDDS and investigated its behavior as a *T*_1_-positive MRI contrast agent. BAC-EDDS contains in each repeating unit two *tert*-amines and four carboxylic groups. It has been prepared by the aza-Michael polyaddition of ethylendiaminodisuccinic acid (EDDS) with 2,2-bis(acrylamido) acetic acid (BAC) (see Scheme 1). BAC-EDDS has been fully characterized by NMR spectroscopy and size exclusion chromatography (SEC).

The Gd-BAC-EDDS complex was obtained by reacting BAC-EDDS in water with GdCl_3_, obtaining, after purification and lyophilization, a soft white solid. The chelate thermodynamic stability constants were estimated using a simplified model system (Agly-EDDS) mimicking the structure of a single BAC-EDDS repeat unit, successfully synthesized by reacting N-acryloylglycine with EDDS in a 2:1 ratio. The Gd-Agly-EDDS complex showed good stability, comparable to that of the Gd(III) complex with diethylenetriamine pentaacetic acid (Gd-DTPA) [22].

To characterize the efficiency of the Gd-BAC-EDDS polymer complex as an MRI contrast agent, the relaxivity profiles (*r*_1_ and *r*_2_) have been measured and interpreted using a model derived from the Solomon–Bloembergen–Morgan theory. Finally, phantom MRI images at 8.5 MHz were acquired showing the great superiority of this polymeric Gd-chelate compared to the commercial contrast agent Magnevist^®^.

## 2. Results and Discussion

### 2.1. Synthesis and Characterization of BAC-EDDS Homopolymer

**Scheme 2:**
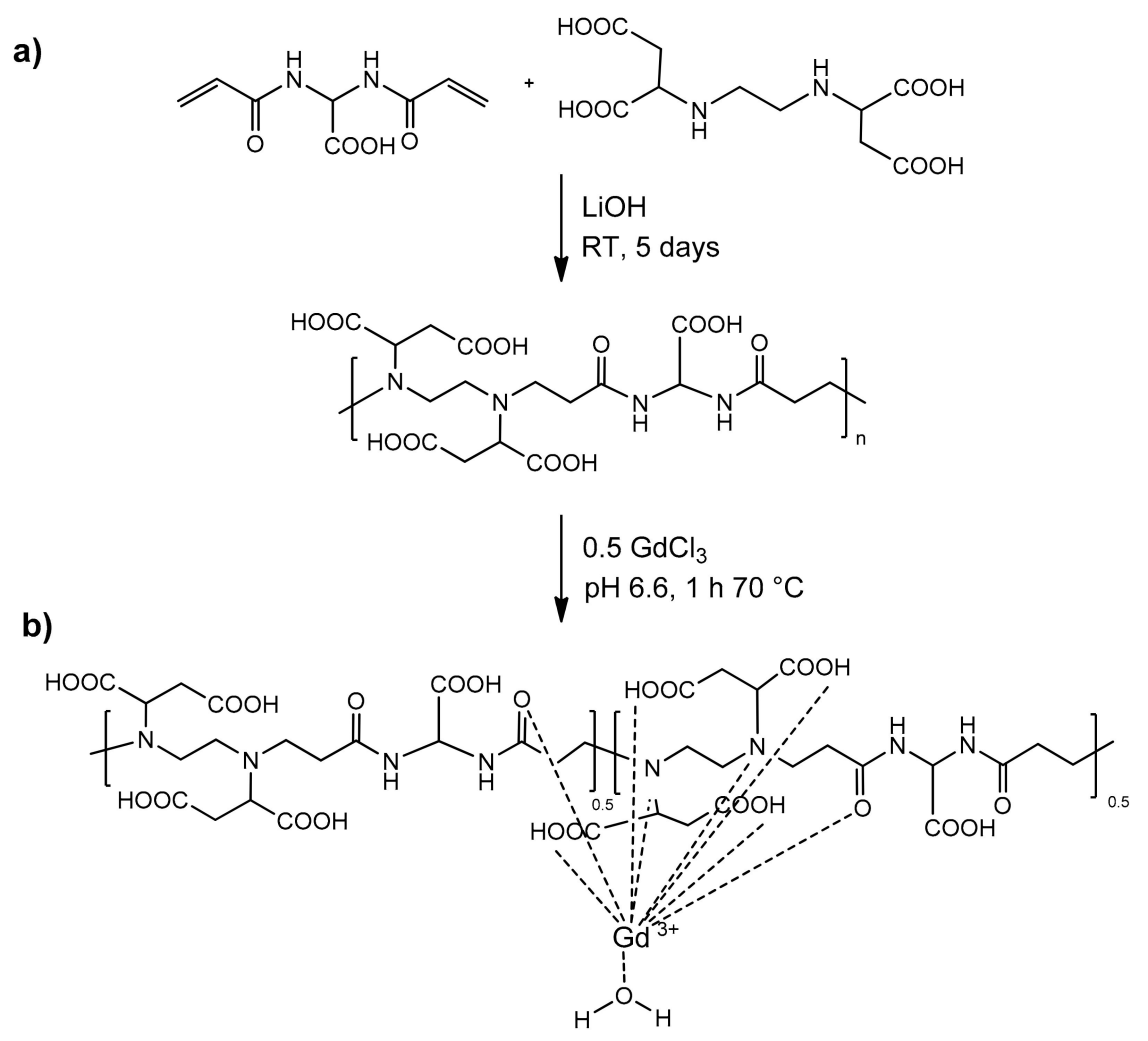
Synthesis of (**a**) BAC-EDDS by the aza-Michael addition of BAC and EDDS and (**b**) the corresponding reaction of Gd(III) to give Gd-BAC-EDDS. The depicted coordination sphere is one representation only, with the COOH groups of BAC moiety possibly also available for interacting with Gd^3+^ ion.

BAC-EDDS was synthesized by the aza-Michael polyaddition of EDDS and 2,2-bis(acrylamido) acetic acid (BAC) in a 1:1 molar ratio, as illustrated in Scheme 2a. The reaction was carried out in water solution for 4–5 days at room temperature, with a reactant concentration 30% *w*/*w* and at pH ~10, adjusted by the addition of lithium hydroxide monohydrate.

The raw polymer was purified by ultrafiltration through a 5000 Da cutoff membrane. SEC analysis showed a number-average molecular weight of Mn = 6800 and a weight-average molecular weight of Mw = 8800 (polydispersity index PD = 1.3), while the elemental analysis suggested that after lyophilization the polymer was tetra-hydrated (see Table 1).

BAC-EDDS bears several possible coordination sites per repeat unit, namely, four carboxylates and two *tert*-amines of the EDDS moiety, and two amides (see Scheme 2) together with a fifth carboxylate of the BAC moiety. The presence of such a number of ionizable sites gives the polymer considerable water solubility.

The FTIR spectrum of BAC-EDDS (Figure S1) shows the typical strong bands at 1720 cm^−1^, attributable to the COOH stretching, and 1640 cm^−1^ due to the amide II band. The band at 1398 cm^−1^ together with bands in the range 3100–2800 cm^−1^ are attributable to CH vibration modes of methylene groups. The UV–Vis absorption spectrum (Figure S2) showed two bands with λ_max_ at 200 and 220 nm attributable to the typical amide *π*→π* and n→π* transitions.

**Figure 1:**
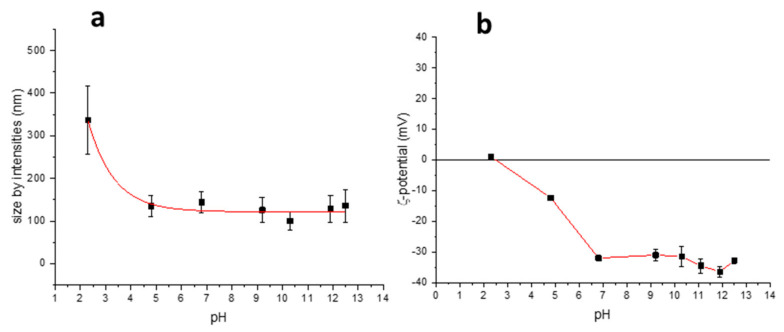
(**a**) Hydrodynamic diameters, and (**b**) ζ-potential as a function of pH, of the polymer BAC-EDDS in water.

As stated for other linear PAAs [23,24,25], BAC-EDDS also forms aggregates in water solution. DLS measurements showed nanoaggregates with a distribution of the hydrodynamic diameter dependent on pH, passing from 340 nm at pH 2 to approximately 150 nm at pH 5 (Figure 1a). This behavior was explained in terms of the pH dependence of the BAC-EDDS solubility. In fact, this polymer at pH <3 begins to aggregate and tends to precipitate, due to the protonation of the carboxylic groups. The trend of the ζ-potential in the same pH range (Figure 1b) showed that the surface charge of the polymer nanoaggregate changed from about 0 to −30 mV when increasing the pH from 2 to 7. This trend could be explained by considering the IP < 2 of BAC-EDDS, as determined by potentiometric titration with the De Levie method [26] as well as by Hyperquad software [27] (vide infra).

### 2.2. Solution NMR Characterization of BAC-EDDS

**Figure 2:**
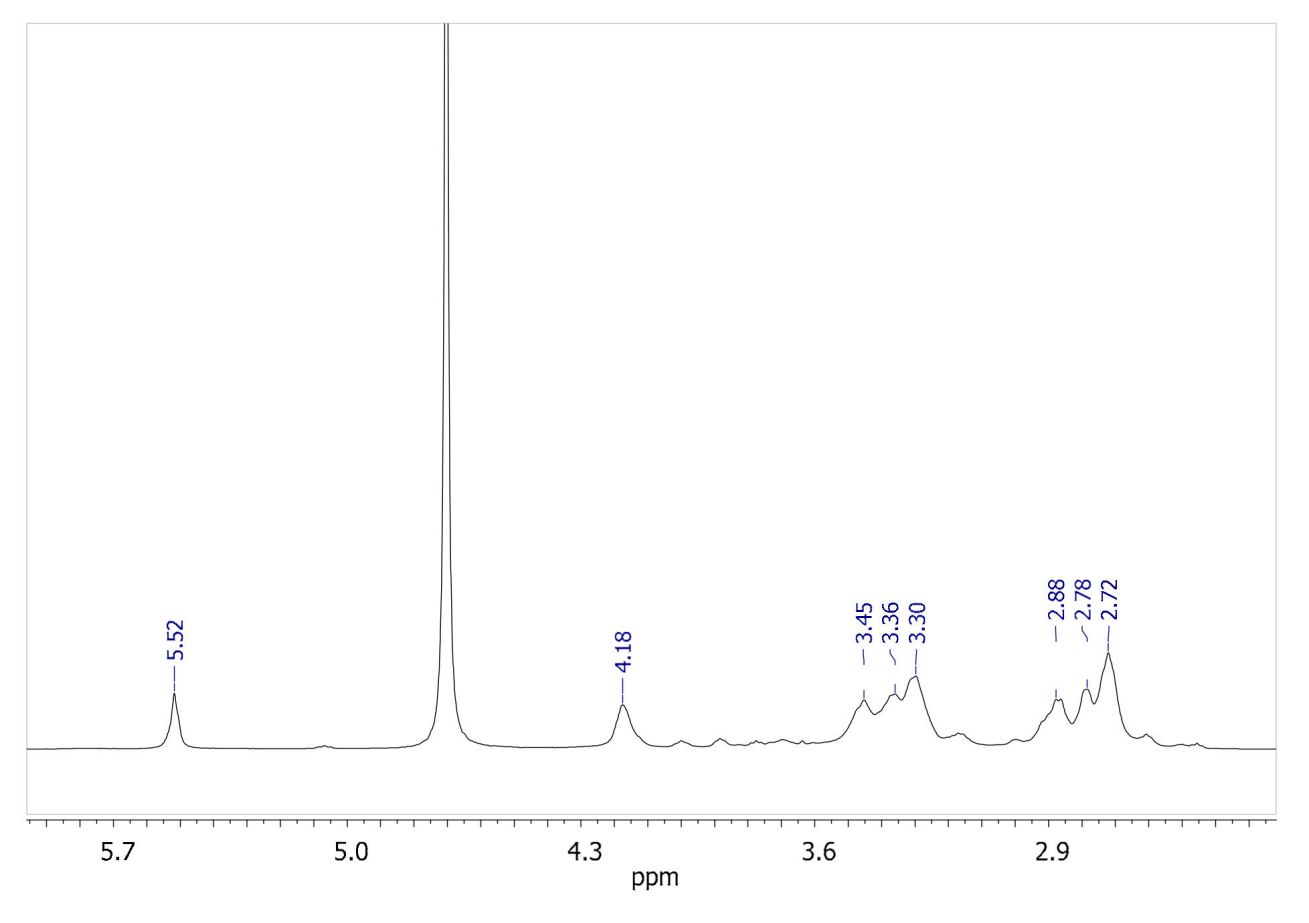
^1^H NMR of a sample of EDDS-BAC polymer in D_2_O (300 K, 9.4 T).

**Figure 3:**
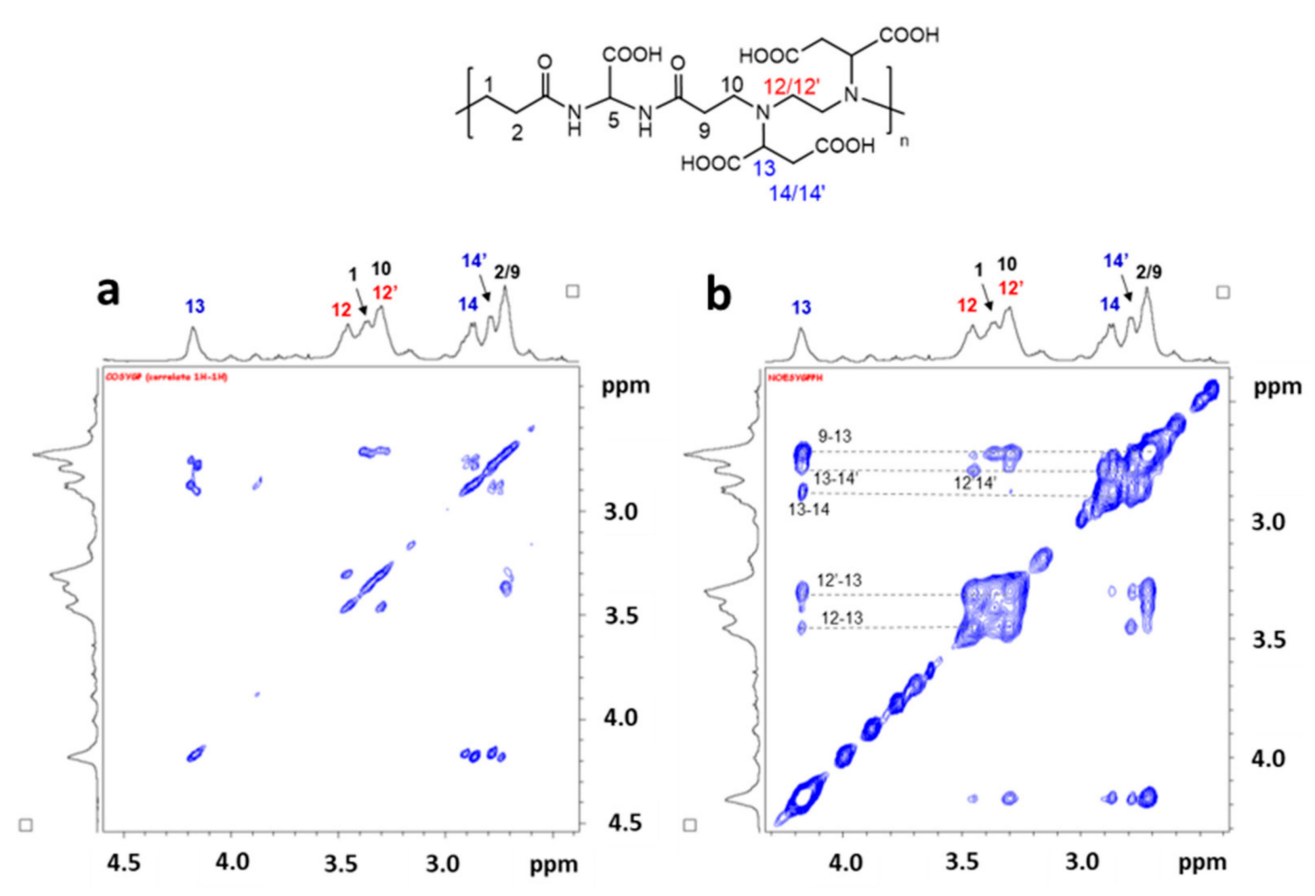
Aliphatic region of 2D NMR: (**a**) ^1^H COSY and (**b**) ^1^H NOESY experiments on a sample of BAC-EDDS homopolymer (D_2_O, 300 K, 9.4 T, pH = 4.4).

Figure 2 shows the room temperature ^1^H-NMR of a sample of BAC-EDDS in D_2_O at pH 4.4. At this pH value, not only the signals of carboxylic protons but also the amide ones are not visible due to fast exchange on the NMR time scale with deuterated water. All the aliphatic signals are broad and poorly resolved due to the nanometric sizes of the polymer. Nevertheless, they were attributed by 2D ^1^H COSY (Figure 3a) and ^1^H NOESY (Figure 3b) NMR experiments.

The singlet lying at 5.52 ppm is attributable to CH(5) between the two amide groups of the original bisacrylamide (for the numbering, see the chart in Figure 3). As this proton is both spatially and scalarly isolated from all the other polymer protons, we identified in the signal lying at 4.18 ppm a new starting point, with this resonance shifted to lower fields compared to the other aliphatic signals, and thus attributable to the two CH protons in position (13) at 4.16 ppm. The ^1^H COSY map shows two cross-peaks between the diagonal peak of CH(13) and two signals centered at 2.79 and 2.87 ppm, which in turn are coupled to each other. This scalar correlation pattern unequivocally allows attributing these two last signals to the diasterotopic protons of the CH_2_ in positions (14/14′). The last two CH_2_ belonging to the EDDS fragment (CH_2_(12/12′)) give rise to the two signals (scalarly coupled to each other) centered at 3.45 and 3.30 ppm or vice versa. Finally, the two CH_2_ (2,9) are accidentally overlapped at 2.72 ppm by a simple comparison with the chemical shift of other similar polyamidoamines such as the homopolymer ISA23 ([23] and refs therein) containing the same bisacrylamide. This last signal is scalarly coupled to the resonance at 3.37 ppm and, consequently, it is easily attributed to the CH_2_ (1,10). The whole assignment was confirmed by the dipolar correlations shown in the 2D ^1^H NOESY (Figure 3b). In this experiment, dipolar correlations (other than the scalar correlations already present in the 2D COSY experiment as scalar couplings) are highlighted by dotted lines in the 2D map (Figure 3b), confirming the spatial closeness of these proton couples, thus supporting their attribution.

### 2.3. Formation of the Polymer Complex

The first interaction of Gd with BAC-EDDS is very quick to occur, also at room temperature, even if, most likely, the first interaction leads to makes the kinetic product, and the formation of the thermodynamic chelate takes more time or needs to be conducted at a higher temperature. Moreover, to avoid the precipitation of the polymeric complex, some precautions have to be taken into account. Indeed, we found that a stoichiometric amount of Gd(III), added one-shot, caused the precipitation of the polymer. To avoid the PAA-Gd precipitation, it is necessary to add Gd(III) in small amounts (see Materials and Methods for details) at a time and strictly maintaining the pH at 7. The purification from the possible Gd(III) excess was effectively carried out by employing the Chelex100 resin (see below).

ζ-potential measurements, carried out at pH ≈ 7 on a Gd-BAC-EDDS sample containing 0.5 equivalents Gd per repeating unit (Figure S3), showed after complexation the expected decrease in the net charge of the polymer complex with respect to the parent polymer, as revealed by the shift in the ζ-potential from −30 ± 10 mV to −20 ± 10 mV. This induced an increase in the size of the aggregates, whose average hydrodynamic diameter passed from 150 to 255 ± 90 nm, as revealed by DLS measurements.

### 2.4. Determination of the Maximum Amount of Gd Complexed by BAC-EDDS Homopolymer by Xylenol Orange

An important aspect to be ascertained about the interaction between the homopolymer and Gd(III) ions relies on the maximum amount of lanthanide ions retained by the chelating moieties. Indeed, *r*_1_ and *r*_2_ nuclear relaxivity values strictly depend on the equivalents of Gd(III) per polymer coil, which contribute to the capability of the polymer complex to act as an effective contrast agent. Since the chelating moiety in this homopolymer is an integrating part of the polymer backbone, it could be expected that not all the repeating units chelate a Gd(III) ion. As a consequence, the Gd(III) equivalents tightly bound to the polymer could be expected to be less than 1 equivalent.

To assess the amount of Gd(III) tightly retained by the polymer, we carried out a titration procedure based on the use of the dye Xylenol Orange as a colorimetric indicator and by the use of the UV–Vis absorption spectroscopy, as reported in the literature [28]. Xylenol Orange assumes different colors according to the environment pH, passing from yellow-orange for pH ≤ 7 to violet at pH > 8, due to the deprotonation of the phenol groups at basic pH. In the presence of Gd(III), its behavior is very similar to the pH-dependence one. Indeed, when the two iminodiacetic groups together with the deprotonated phenol interact with the cationic lanthanide, the complex assumes a violet color. Hence, it is extremely important to carry out the titration buffering of the solution at acidic pH. For this purpose, the titration was carried out at pH = 5.4 by using an acetic buffer solution. Thanks to the relation existing between the free Gd(III) mmol and the variation in the ratio of the intensity of the two characteristic Xylenol Orange absorption bands, centered at 578 and 434 nm, the amount of free Gd(III) is easily determined (Figure S4). To this purpose, it is necessary to build up a calibration curve by using standard solutions of Gd(III) (Equation (1), see Materials and Methods for details).

(1) $$Gd{\left(III\right)}_{free}\;mmol=a+b\;\cdot \;\frac{A_{578\;nm}}{A_{434\;nm}}\;$$

Using this spectrophotometric analytical method, it was possible to ascertain the ability of the PAA to bind Gd(III) ions and determine the amount of free Gd(III) deriving from equilibrium with the polymer complex PAA-Gd. As a result, the spectrophotometric titration indicated that the maximum percentage of Gd that is stably bonded by the polymer is about 52% on a molar basis, ensuring the stable coordination of 0.5 equivalents of Gd per EDDS groups present in each PAA repeating unit.

### 2.5. PAA-Gd Purification from the Excess of Unbound Gd(III)

If the spectrophotometric analysis based on Xylenol Orange reveals an amount of free Gd(III) higher than 0.3% mol/mol, the adduct must be purified before being considered acceptable for an administration. Different purification methods were taken into account such as ultrafiltration, dialysis and treatment with the resin Chelex100. Both dialysis with a 5000 kDa cutoff membrane and the Amicon ultrafiltration under a nitrogen flux with a 3000 kDa cutoff membrane were unable to effectively separate the Gd(III) excess from the polymer, likely due to the weak but effective interactions that make the passage of the Gd ions through the membrane pores unfeasible. Additionally, an attempt to selectively precipitate Gd ions as hydroxide failed as Gd(OH)_3_ formed a gel-like matrix that even by centrifugation was not easily separable from the supernatant containing the soluble polymer complex. Finally, we found that very effective removal of Gd ion excess was achieved using the resin Chelex100, which is made of a styrene and divinylbenzene copolymer functionalized by iminodiacetate groups able to chelate metallic ions. This makes Chelex100 a fairly acidic ion-exchange resin that operates as a cationic resin at basic, neutral and weakly acid pH values, whilst at very low pH it behaves as an anionic exchanger. The treatment of the PAA-Gd with Chelex100 in suspension for a few minutes removed most of the free Gd(III) such that the free Gd %mol/mol went down below the maximum allowed limit.

### 2.6. Determination of the Gd Amount in the PAA-Gd Complex by Bulk Magnetic Susceptibility (BMS) Shift Measurements

Evans’s method consists of the determination of the susceptibility by measuring the BMS-shift (∆χ) that the inert diamagnetic *tert*-butanol undergoes in the presence of a paramagnetic species. Indeed, there exists the following relation that correlates the BMS-shift to the Gd^3+^ concentration according to Equation (2), where *s* is a parameter depending on the sample shape and its position in the magnetic field (in our case this is equal to 1/3), *T* is the temperature in Kelvin and *μ_eff_* is the effective magnetic moment of the lanthanide, which for Gd(III) is equal to 7.94 [29].

(2) $$\Delta \chi =\left(\frac{4\pi \left[Gd^{3+}\right]s}{T}\right)\cdot {\left(\frac{\mu_{eff}}{2.84}\right)}^{2}\cdot 10^{3}$$

In practice, a 1% *v/v tert*-butanol water solution is inserted in the inner and outer part of a coaxial NMR tube, while only in one of the two compartments a solution of Gd(III) of unknown concentration is added. Through this method, a sample of Gd-BAC-EDDS treated with Chelex100 was used to measure the final Gd(III) amount retained by the PAA. Two successive aliquots of the polymeric complex were added to the coaxial tube and the *tert*-butanol methyl chemical shift difference (∆χ) in the two compartments was measured (Figure S5). Applying the reverse formula of Equation (2) for the [Gd^3+^] determination, it was estimated that ca. 50% of Gd was stably chelated by the PAA, in agreement with the spectrophotometric result. The results achieved by this method were also in agreement with the ICP-AES measurements, as also already reported in the literature [30].

### 2.7. ^17^O NMR Measurements for the Determination of q Number

The water nuclear relaxation rates strictly depend on the number of inner-sphere water molecules directly coordinated to the gadolinium ion. A simple technique that enables quantifying the number *q* of coordinated molecules is based on the Ln(III)-induced ^17^O shift [31]. By carrying out a titration of the solution containing a known amount of ligand with known solutions of Gd(III), it is possible to follow the shift induced in the signal of ^17^O of water as a function of the growing concentration of the lanthanide salt. If the interaction between the Gd(III) and the ligand is tight enough, the plot of the observed ^17^O water shift vs. the Gd(III) concentration gives a straight line whose slope is proportional to the hydration number of Gd(III) complex. Hence, by using as a reference complex the known [Gd(H_2_O)_9_]^3+^, the slope ratio of the two curves directly provides the *q* number for the complex of interest.

Analyzing the induced shift for a solution of pure D_2_O and a solution of BAC-EDDS of known concentration (Figure S6), we found that the *q* number for the Gd-BAC-EDDS complex is equal to 1.

### 2.8. The Synthesis of a Monomer Model

**Scheme 3:**
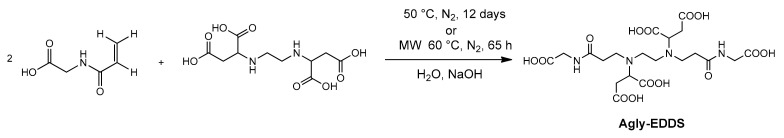
Synthesis of the molecular model Agly-EDDS.

Due to the complexity of the polymer complex Gd-BAC-EDDS, we preferred to estimate the stability constants on a molecular complex constituted by a ligand capable as much as possible of representing the repeat units of the homopolymer. Hence, for this purpose, we prepared the new ligand Agly-EDDS, which is represented in Scheme 3. Acryloyl glycine was firstly synthesized by following a literature method [32] and then reacted with EDDS in a molar ratio 2:1 to give a Michael addition of the two secondary amines of EDDS on the double bond of two acryloyl glycines (Scheme 3). To achieve the completeness of the reaction in a reasonable time, the reaction needed (as in the case of the synthesis of the homopolymer BAC-EDDS, see below) to be conducted in a very concentrated solution (at least 30% *w*/*w* of reactants), by warming at 50 °C and adjusting the pH at 10. Due to the high concentration of double bond species, the reaction was conducted in the dark and under N_2_, for up to 12 days. Alternatively, the synthetic procedure was also carried out by heating through microwave irradiation of the mixture, and in this case, the reaction lasted 65 h only. After the crude product was purified by extracting the unreacted acryloyl glycine with ethyl acetate (see Materials and Methods for details), a full NMR characterization of the product (Agly-EDDS) was carried out (see Supplementary Materials Figures S7–S9). The nature of the product was also ascertained by elemental analysis and mass spectrometry (Figure S10).

### 2.9. Determination of the pKa of Agly-EDDS and BAC-EDDS

The pK_a_ values of the carboxyl and *tert*-amino functions of both Agly-EDDS and BAC-EDDS were determined by potentiometric titration following the De Levie approach [33], as previously reported for other linear polyamidoamines, and using the Hyperquad software. The potentiometric data were confirmed, in the case of Agly-EDDS, by performing a ^1^H NMR titration which allowed for assigning the protonation site to each pK_a_ value.

The De Levie method consists of a simulation based on the systematic treatment of the several equilibria that are contemporarily active in the system and enables solving the analytical problem, by iteratively adjusting, at the same time, the ionic strength and, accordingly, the activity coefficients. Contrarily to the traditional methods of the data treatment, which apply a series of approximations, De Levie uses a graphical method to describe all the equilibria processes that need to be considered. To do so, the mass and charge balances are taken into account, together with the protonic balance, which expresses the balance between the lost and gained protons. The De Levie method consists in finding one general equation able to analytically describe each entire acid–base titration process. Hence, it is possible to obtain a simulated curve, in which the titrant volume is expressed as a function of the pH (or H^+^ concentration), in the opposite manner to the traditional methods, where the unknown parameter is the pH expressed as a function of the titrating volumes.

To obtain the general equation in which the titrating volume is the unknown species, we have to consider the charge balance and the α_i_ dissociation grades of all the species present in solution, eventually obtaining the following equation:

(3) $$\frac{V_{a}}{V_{b}}=\frac{\sum F_{a}\cdot \;C_{a}-\Delta }{\sum F_{b}\cdot C_{b}+\Delta }$$

where *V_a_* is the volume of the polyprotic species to be titrated, *V_b_* is the volume of the added titrating base, *C_a_* and *C_b_* are their concentrations, respectively, and Δ is defined as the sum of [H^+^] + [OH^−^]. *F_a_* and *F_b_* are the dissociation functions (that are polynomials written as a sum of the acid dissociation degrees α_i_, each with its numerical coefficient dependent on the proton associations and dissociations of the species) of the molecules of acid and base in examination (for the analytical expressions of Agly-EDDS and BAC-EDDS and the whole treatment, see the Supplementary Materials). Thus, the experimentally collected titration data are superimposed with the simulation curve in a calculation sheet program, based on the equation of the titrating base volume *V_b_* vs. pH, and by following iterative attempts that contemporarily change the several pK_a_ values, it is possible to graphically provide a nice estimation of such pK_a_ values.

**Figure 4:**
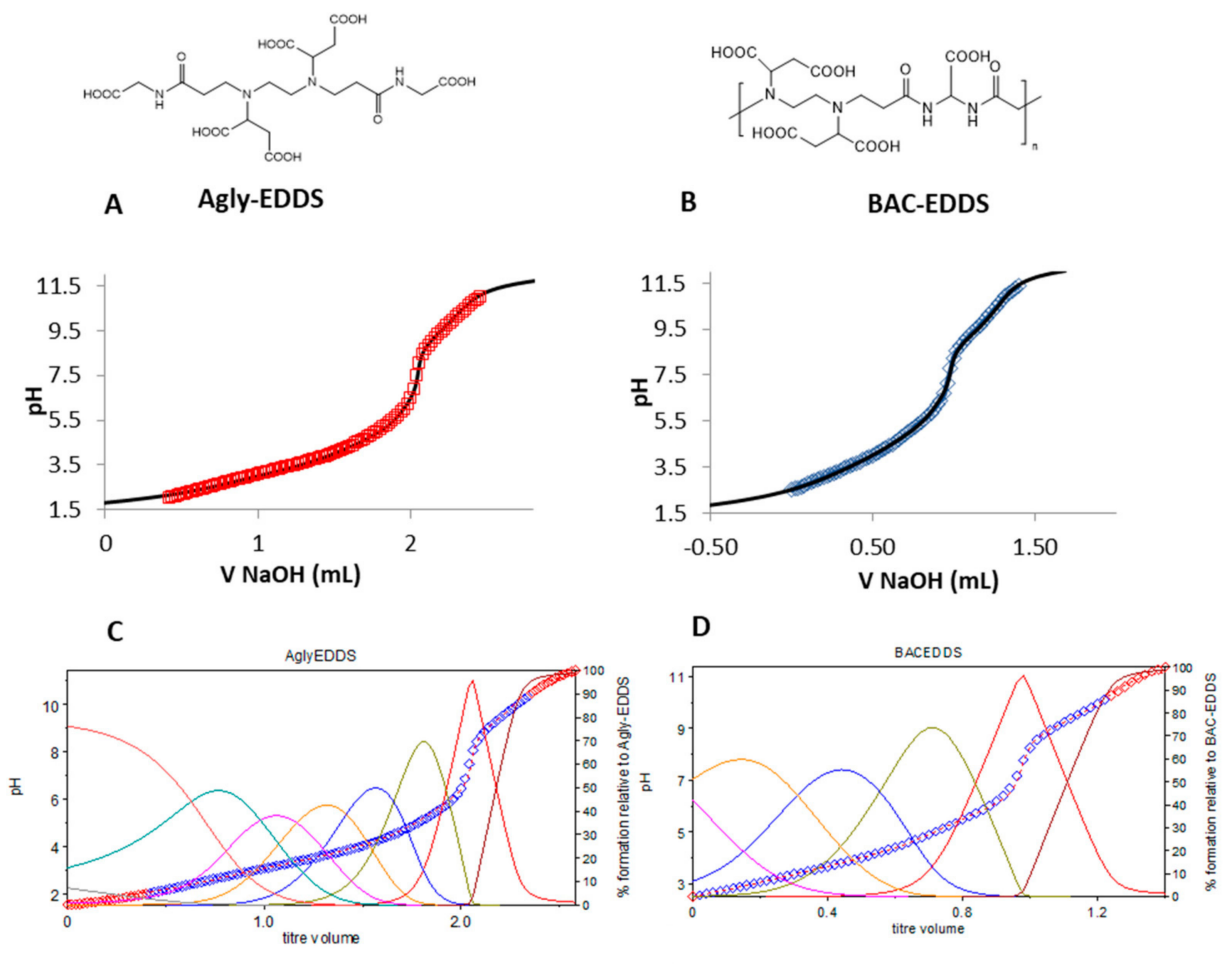
Superposition of the experimental data of the titration curves of (**A**) Agly-EDDS and (**B**) with NaOH and the simulation curves by the De Levie method. (**C**,**D**) Fitting of the same titration curves by Hyperquad software. The colored lines in panels (**C**) and (**D**) represent the speciation of the ligands expressed as % of the different protonated ligands.

In Figure 4, the simulation curves obtained with such a method superimposed on the experimental titration data for the molecule Agly-EDDS and the polymer BAC-EDDS (panels A and B, respectively) are displayed, together with the speciation diagrams (panels C and D) deriving from the estimated pK_a_ by Hyperquad and reported in Table 2. Compared to the BAC-EDDS repeat unit, in Agly-EDDS there is an extra protonation site. For BAC-EDDS polymer, the first two protonation constants were not detected as the polymer at very low pH precipitated, while the pK_a1_ for Agly-EDDS was not estimated because lower than 2, and was hence out of the work range of the employed glass electrode. The estimated pK_a_ values are similar for the two methods used with the exception of the higher pK_a_ value for BAC-EDDS that was lower in the case of Hyperquad fitting than De Levie simulation. The prevailing intervals based on pK_a_s derived from both De Levie and Hyperquad for the two ligands are reported in Figure S10.

In order to assign the pK_a_ to each protonation site, we carried out a ^1^H NMR titration on Agly-EDDS (Figure S12) that revealed the pH range at which the variation in the chemical shift in the resonances of the CH and CH_2_ close to the acidic sites occurs, thus attesting the site at which the deprotonation event occurs as well.

**Figure 5:**
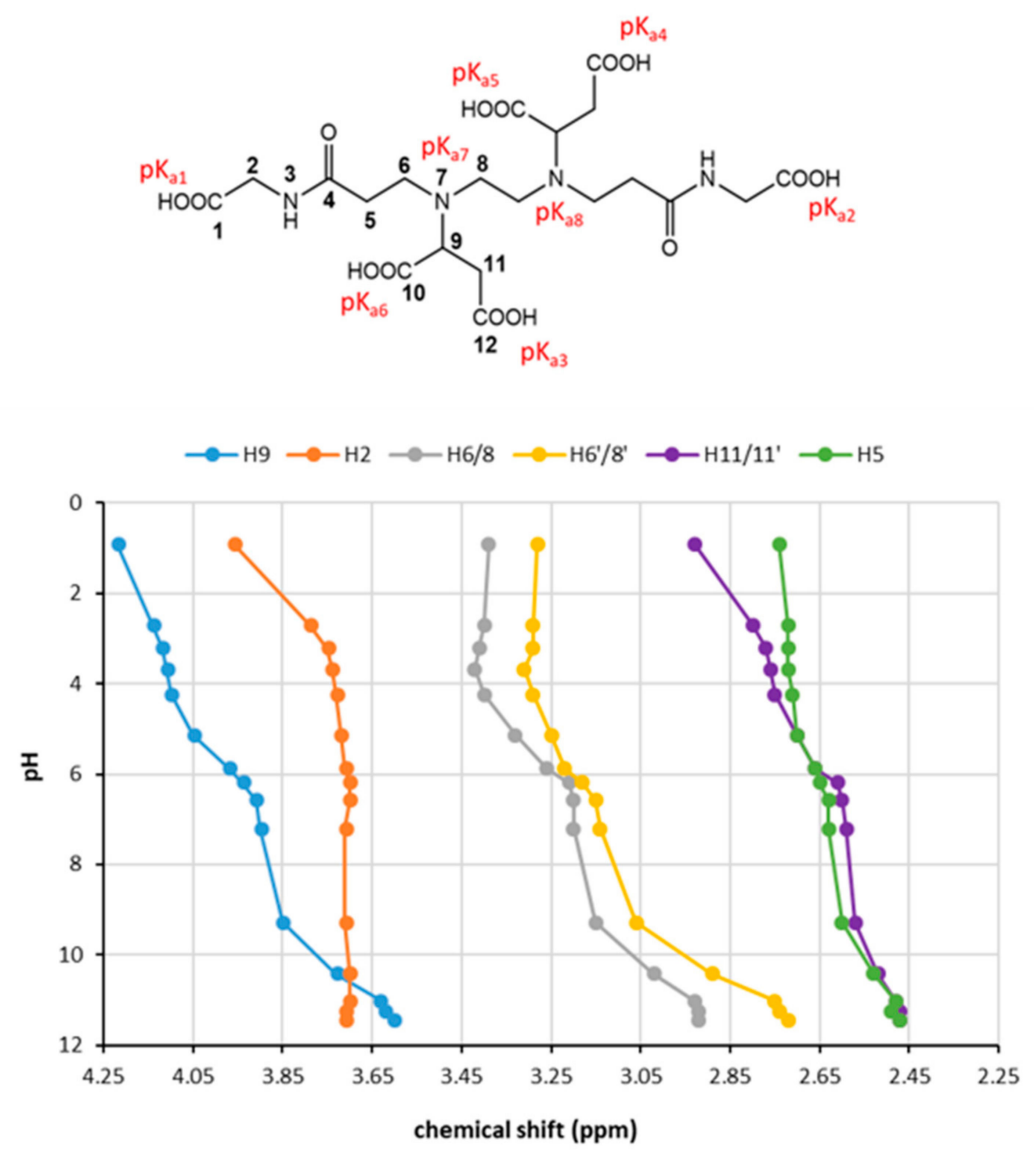
^1^H NMR titration of Agly-EDDS together with a tentative attribution of the pK_a_ to the acid sites.

The first two sites that deprotonate are ascribable to the two COOH (1) of acryloyl moiety that cause an upfield shift in the resonance of the CH_2_ (2). The signals relative to CH_2_ (11/11′) undergo an upfield shift at early pH that indicates a COOH (12) as the next deprotonated site. In the same way, in the pH range 3.8–6.0, it further shifts, indicating the deprotonation of the second COOH (12). Moreover, it is clearly visible that CH (9) undergoes over the whole pH range a chemical shift variation in three successive steps that correspond to the three deprotonation sites—the two COOH (10) and N(7)—close to this alifatic proton. In particular, the two COOH (10) are supposed to deprotonate in the pH range 4–6, while the amine from pH 8.5 to 10.5. This is confirmed by the chemical shift variation observed in the same pH range for CH_2_ (6/6′) and (8/8′). The CH_2_ (6/6′) and (8/8′) undergo in parallel two step-shifts due to the deprotonation of the two tertiary amines, the first one in the range 4 < pH < 6 and the second one for pH > 8.5. The chemical shift variation in these two CH_2_ close to the NR_3_ sites stops at pH ≈ 10.5. The two pH variation intervals are in agreement with the pK_a7_ and pK_a8_ values obtained by the potentiometric titration. In the same pH range CH_2_ (6/6′) and (8/8′) also undergo an important chemical shift variation at higher fields, parallel to that observed for CH (9), showing that this step corresponds to the same deprotonation event. On the basis of these considerations, a tentative attribution of the pK_a_ to the acid sites is reported in Figure 5 (top).

The results obtained with the ^1^H NMR titration experiment are in agreement with the pK_a_ values obtained by the two methods employed.

### 2.10. Thermodynamic Stability Constant for the Gd-Agly-EDDS Complex

**Figure 6:**
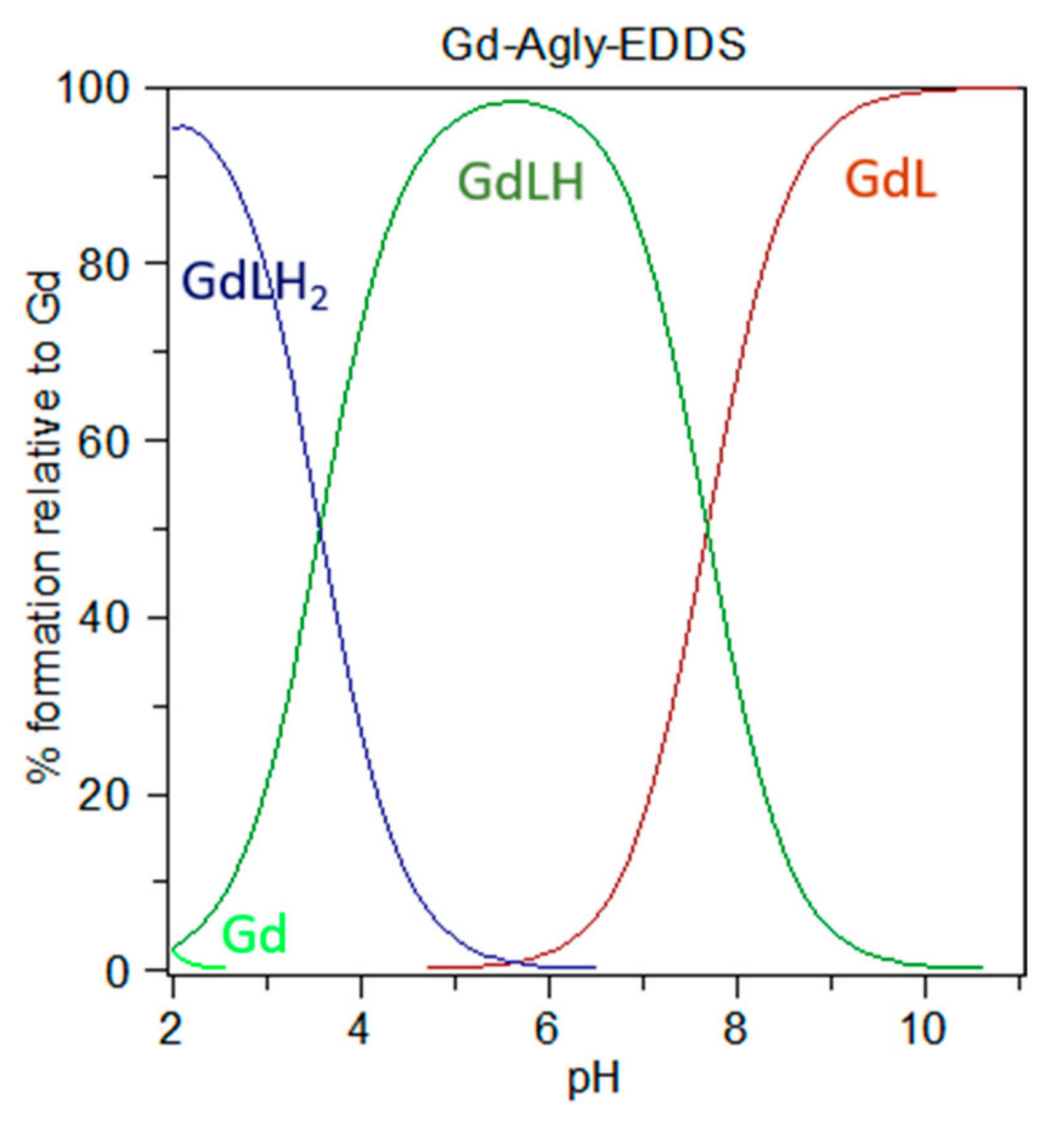
Species distribution diagrams for 1:1 Gd-Agly-EDDS complex (charges of the species are omitted).

The complex stability is a prerequisite that is extremely important for Gd-based MRI contrast agents used at the clinical level, since free Gd^3+^ is highly toxic in vivo. Indeed, the mechanism by which the free gadolinium ions can block the Ca^2+^ binding sites is well known [34]. Nevertheless, the attempts to fit the titration curve of the stable polymer complex containing 0.5 equivalents of the monomer repeat units complexed to Gd^3+^ ions failed, even taking into account many different species in the model, such as GdLOH and several GdLH_i_ (i = 1–5). Hence, to study the thermodynamic stability constants of the polymer complex we took into account the 1:1 molecular complex Gd-Agly-EDDS as a model of what can occur at the polymer complex sites, even though it is clear this is just a fair approximated model. The stability (K_GdL_) and the protonation (K_GdLH_ and K_GdLH2_) constants of the Gd^3+^ complexes with the monomer model Agly-EDDS (1:1) were studied, by fitting the potentiometric titration curves by Hyperquad. The first constant refers to the formation equilibrium Gd^3+^ + L ⇄ GdL (L = Agly-EDDS) and its analytical equation is reported in Table 3. The best fitting was obtained considering the mono- and bi-protonated form of the complex GdLH and GdLH_2_, whose protonation constants K_GdLH_ and K_GdLH2_ are reported in Table 3 (please note that in this notation the real charges are omitted for the sake of clarity). The obtained equilibrium stepwise constants, together with the overall constants (log β), are listed in Table 3. The potentiometric titrations showed that the stability constant for the model complex is log K_GdL_ = 17.43, which is a value comparable to the ones of other Gd(III) complexes based on the use of open DTPA-like ligands [35,36]. On the basis of the obtained stability and protonation constant values, the species distribution diagram of Figure 6 can be drawn. It clearly shows that at physiological pH the prevailing species for the solution containing a 1:1 molar ratio of Gd^3+^/Agly-EDDS is the mono-protonated complex (ca 65%), which presumably protonates at one amine site considering the value of log K_GdLH_, together with the unprotonated GdL complex (ca 35%), while free Gd^3+^ ion is negligible above ca. pH 2.5, where it is present in very low concentrations.

### 2.11. NMR Dispersion Profiles and MRI Contrast Studies

Prior to ex vivo and in vivo experimentation, the consolidated method to test the efficiency of a contrast agent (CA) in contrasting MR images is the evaluation of its longitudinal (*r*_1_) and transverse (*r*_2_) relaxivities as a function of the externally applied static field, the so-called nuclear magnetic relaxation dispersion (NMRD) profiles.

The nuclear relaxivity is defined as

(4) $$r_{i}=\left(\frac{1}{T_{i,\;meas}}-\frac{1}{T_{i,\;dia}}\right)/C$$

where *i* = 1, 2, respectively, refer to the nuclear longitudinal relaxation time *T*_1_ and the nuclear transverse relaxation time *T*_2_. 1/*T_i,meas_* is the nuclear relaxation rate measured on a sample dispersion, 1/*T_i,dia_* is the nuclear relaxation rate of the diamagnetic host solution and C is the concentration of the magnetic center expressed in mmol·L^−1^.

Consequently, high *r*_1_ and *r*_2_ values are generally required for an MRI CA to reach high contrast efficiency [37].

**Figure 7:**
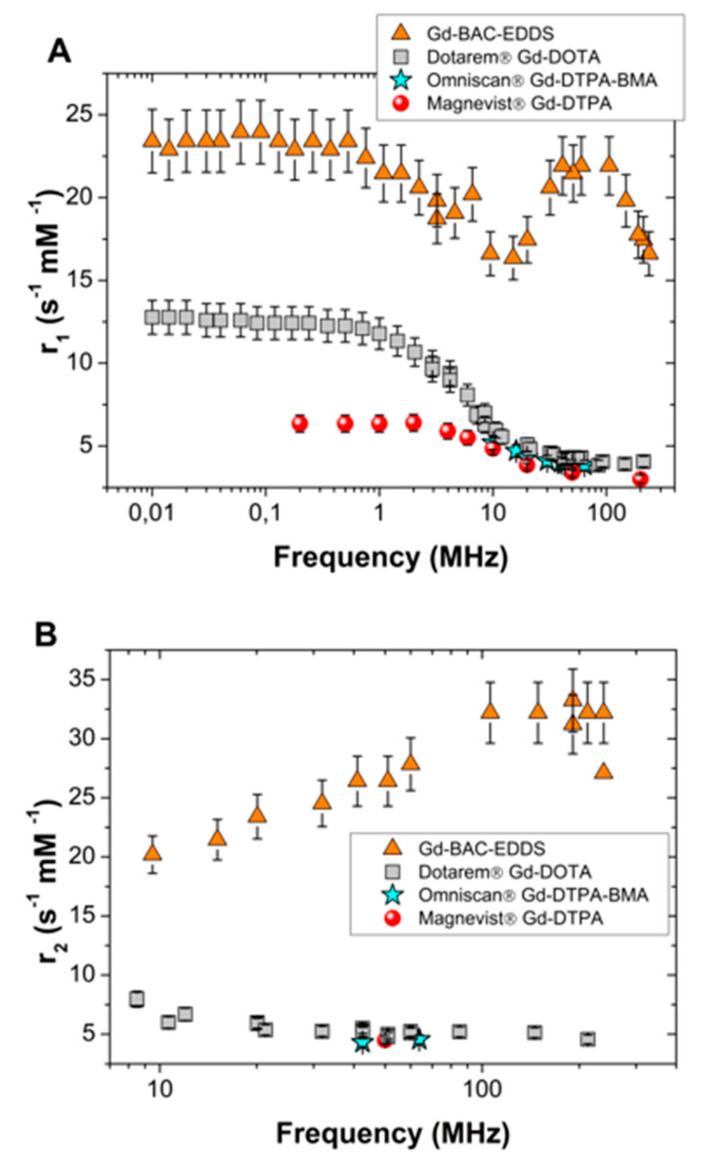
(**A**) Longitudinal (*r*_1_) relaxivity (NMRD profile) at room temperature in the frequency range 0.01 < ν < 240 MHz measured for Gd-BAC-EDDS polymer complex compared to *r*_1_ of three Gd chelate commercial contrast agents measured in similar frequency ranges. (**B**) Transverse (*r*_2_) relaxivity (NMRD profile) at room temperature in the frequency range 10 < ν < 240 MHz measured for Gd-BAC-EDDS polymer complex compared to *r*_1_ of three Gd chelate commercial contrast agents.

As clearly shown by Figure 7, both *r*_1_ and *r*_2_ values of Gd-BAC-EDDS are 2–4 times higher than the relaxivity values of commercial CAs at corresponding frequencies, showing a better ability of Gd-BAC-EDDS to increase the nuclear relaxation rates of the host solution.

Moreover, the shape of *r*_1_ NMRD profile of Gd-BAC-EDDS presents a maximum at 75 MHz that differentiates it from the commercial CAs and accounts for the different molecular dimensions of our system with respect to commercial compounds. Indeed, the increase in dimensions slows down the rotational dynamics of the molecular system and explicitly influences its relaxivity at high frequencies [12].

Therefore, in order to extrapolate the dynamical parameters of interest for the characterization of Gd-BAC-EDDS as an MRI contrast agent, *r*_1_ data of Gd-BAC-EDDS were fitted with a model derived from the Solomon–Bloembergen–Morgan theory (SBM) [38,39].

The hyperfine dipolar interaction that causes the relaxation enhancement in Gd(III) paramagnetic aqueous dilute solutions can be split into three main contributions (the scalar and the Curie contributions can be neglected for Gd(III) complexes): inner-sphere (IS), second-sphere (SS) and outer-sphere (OS) [37]. This differentiation originates from the intra- or inter-molecular nature of the interactions between the nuclear spin of the protons of the water molecules (the solvent) and the electronic spins of the Gd(III) complex (the paramagnetic species). The inner-sphere water molecules reside in the first-coordination sphere, directly bound to the paramagnetic center; the second-sphere water molecules (few) are close to the functional groups of the ligand, without forming a complete spherical shell around the complex; the outer-sphere water molecules correspond to the bulk ones.

The overall relaxivity *r*_1_ is then given by:

(5) $$r_{1}=r_{1}^{IS}+r_{1}^{SS}+r_{1}^{OS}$$

The inner-sphere contribution of the longitudinal relaxation rate *R_1,IS_* = *r_1_^IS^ C* is expressed as follows:

(6) $$R_{1,IS}=fq\frac{1}{T_{1m}+\tau_{m}}$$

with

(7) $$\left(\frac{1}{T_{1m}}\right)=\frac{2}{15}\;{\left(\frac{\mu_{0}}{4\pi }\right)}^{2}\;\frac{\gamma_{I}^{2}g^{2}\mu_{B}^{2}}{r^{6}}S\;\left(S+1\right)\left[7\frac{\tau_{c2}}{1+\omega_{S}^{2}\tau_{c2}^{2}}+3\frac{\tau_{c1}}{1+\omega_{I}^{2}\tau_{c1}^{2}}\right]$$

where *f* is the ratio between the concentration of the paramagnetic species and the water (*f* = *C*/55,500), *q* is the number of coordinated water molecules (hydration number), *T_1m_* is the proton relaxation time of the coordinated water, *τ_m_* is the water exchange time of the coordinated water molecules with the bulk, *γ_I_* is the gyromagnetic ratio of the observed nucleus, *g* is the electron g-factor, *µ_B_* is the Bohr magneton, *r* is the distance between the paramagnetic ion and the observed nucleus, *S* is the spin quantum number, *µ_0_* is the vacuum magnetic permeability, *ω_I_* and *ω_S_* are the nuclear and electron angular precession frequencies. The correlation times *τ_ci_* modulating the dipolar interaction are given by

*τ_ci_*^−1^ = *τ_m_*^−1^ + *τ_r_*^−1^ + *T_ie_*^−1^     with *i* = 1, 2 (8)

where *τ_r_* is the rotational correlation time of the complex and *T_ie_* are the electronic relaxation times. In the Redfield limit and for metal complexes with *S* ≥ 1, the electronic relaxation rates (1/*T_ie_*) are usually written by taking into account the zero-field splitting (ZFS) interaction as follows:

(9) $$\left(\frac{1}{T_{1e}}\right)=2\tilde{C}\left(\frac{1}{1+\omega_{S}^{2}\tau_{v}^{2}}+\frac{4}{1+4\omega_{S}^{2}\tau_{v}^{2}}\right)$$

(10) $$\left(\frac{1}{T_{2e}}\right)=\tilde{C}\left(\frac{5}{1+\omega_{S}^{2}\tau_{v}^{2}}+\frac{2}{1+4\omega_{S}^{2}\tau_{v}^{2}}+3\right)$$

with $\tilde{C}$ = 1/50 Δ^2^ *τ_v_* [4 *S* (*S +* 1) − 3], where Δ^2^ is the mean squared fluctuation of the ZFS and *τ_v_* is the ZFS modulation correlation time.

The expressions for the second-sphere contribution are similar to the inner-sphere ones substituting the corresponding parameters for the second-sphere water molecules (primed parameters): *q*’, *τ_m_*’, *r*’.

The equations for the outer-sphere relaxation rate of bulk water molecules *R_1,OS_* = *r*_1_*^OS^ C* are given by:

(11) $$R_{1,\;\;OS}=\frac{32\pi }{405}\;{\left(\frac{\mu_{0}}{4\pi }\right)}^{2}\frac{N_{A}C}{dD}\;\gamma_{I}^{2}g^{2}\mu_{B\;}^{2}\;S\left(S+1\right)\;\left[7\;j_{2}\left(\omega_{S}\right)+3\;j_{1}\left(\omega_{I}\right)\right]$$

where *N_A_* is the Avogadro number, *d* is the distance of minimum approach for bulk water molecules to the paramagnetic center, *D* is the relative self-diffusion constant and *j_k_*(*ω*) is the spectral density function for the dipolar interaction

(12) jkω=Re 1+z/41+z+4 z2/9+z3/9

with $z=\sqrt{i\omega \tau_{D}+\tau_{D}/T_{ke}}$ (*k* = 1, 2), and $\tau_{D}=d^{2}/D$ is the translational correlation time.

**Figure 8:**
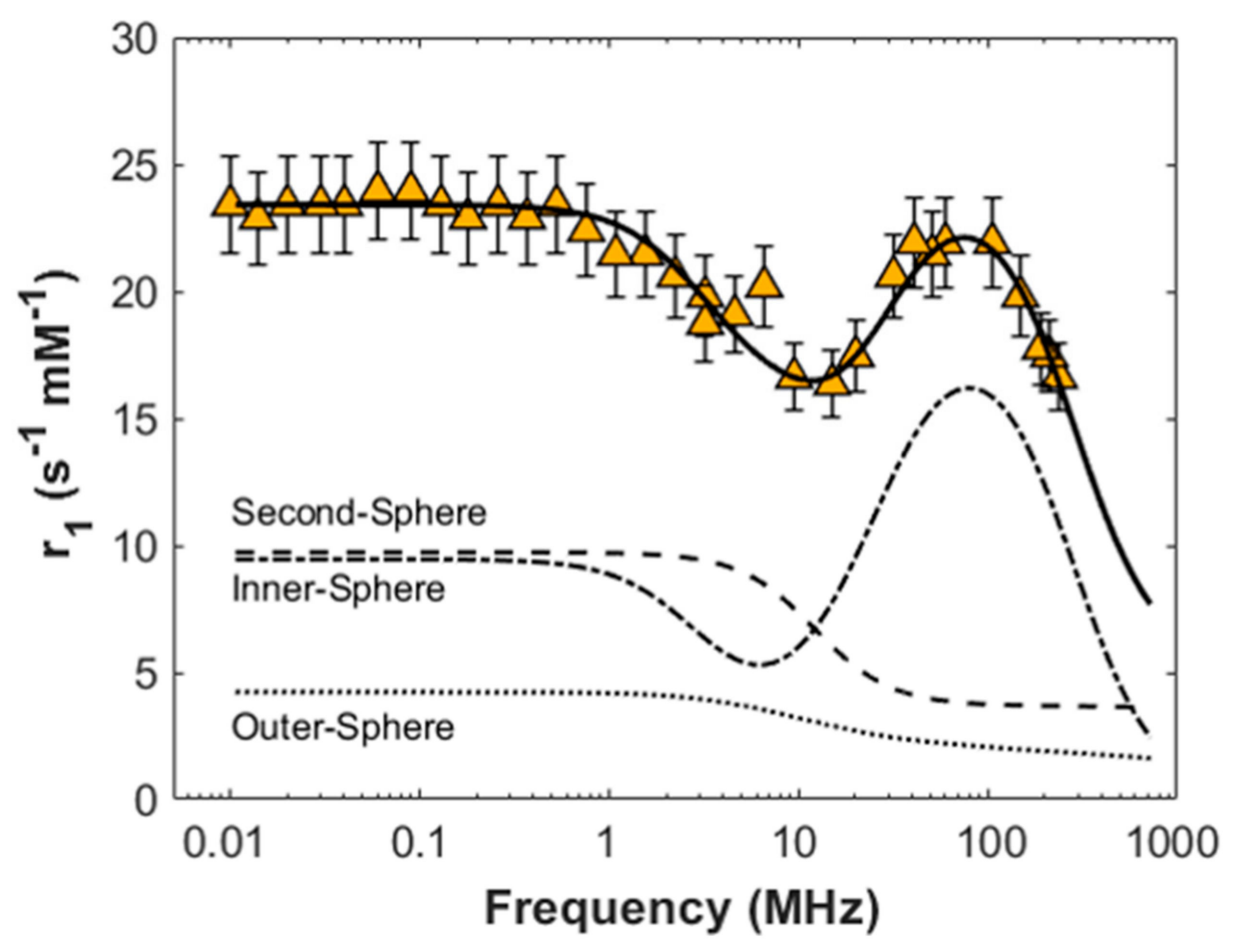
Fit of the *r*_1_ NMRD profile data with the SBM model equations. The different contributions to the longitudinal relaxivity (inner-, second- and outer-sphere) are also highlighted. Best fit parameters: *q* = 1 (fixed), *r* = 3.0 Å (fixed), *τ_r_* = 0.66 ± 0.05 ns, *τ_m_* = 254 ± 38 ns, *q’* = 5.8 ± 0.2, *r’* = 3.2 ± 0.1 Å, *τ_m_*’ = 26 ± 5 ps, Δ = 0.040 ± 0.004 cm^−1^, *τ_v_* = 15 ± 2 ps. The following assumptions were made: *q =* 1, *r =* 3.0 Å, *d* = 3.5 Å and *D* = 2.3 × 10^−9^ m^2^ s^−1^.

The fitted data are reported in Figure 8.

We used as adjustable parameters *τ_r_*, *τ_m_*, *q’*, *r’*, *τ_m_*’, Δ^2^ and *τ_v_*, assuming *q* = 1 (in agreement with ^17^O measurements), *r* = 3.0 Å, *d* = 3.5 Å and *D* = 2.3 × 10^−9^ m^2^ s^−1^ (i.e., the relative diffusion coefficient of pure water) [40,41].

The observed peak *r*_1_ = 22 ± 2 s^−1^ mM^−1^ at 75 MHz is consistent with the long rotational correlation time obtained from the fit, *τ_r_* = 0.66 ± 0.05 ns, while the values of *τ_m_* = 254 ± 38 ns, Δ = 0.040 ± 0.004 cm^−1^ and *τ_v_* = 15 ± 2 ps are similar to the ones already reported for Gd-based contrast agents [40].

As can be seen in Figure 8, the second-sphere contribution, due to almost six water molecules (*q*’ = 5.8 ± 0.2) at an average distance from the metal ion of *r*’ = 3.2 ± 0.1 Å characterized by a fast exchange with the bulk (*τ_m_*’ = 26 ± 5 ps), plays a non-negligible role in the relaxation enhancement, being comparable to the inner-sphere one at low frequencies [12].

**Figure 9:**
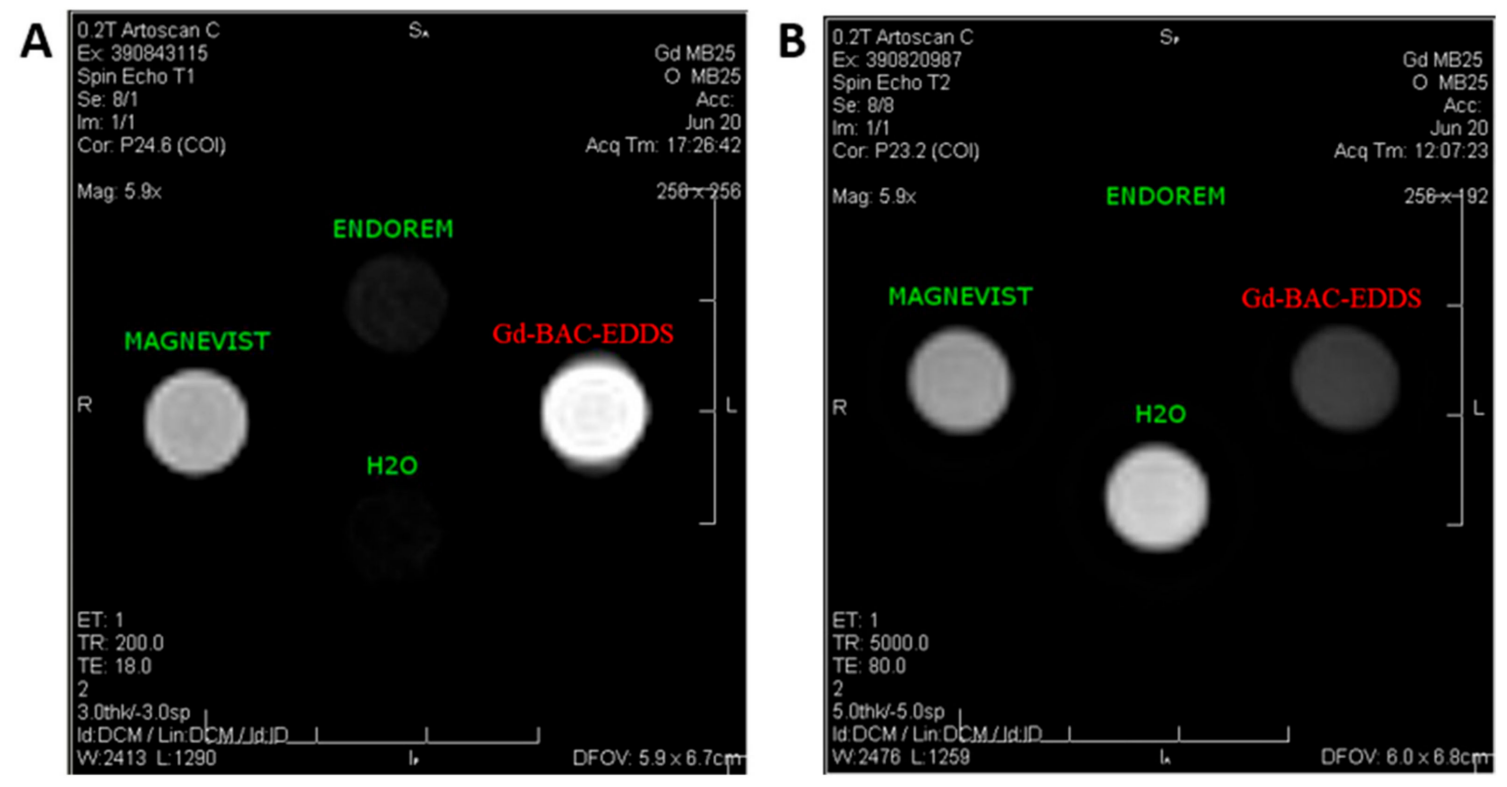
In vitro contrast efficiency of Gd-BAC-EDDS as MRI agent. MRI images of vials containing Gd-BAC-EDDS, Magnevist^®^ and Endorem^®^ at the same concentration (~0.85 mM), and ultrapure water, obtained by an Artoscan (by Esaote SpA) imager at 8.5 MHz at room temperature by means of a high-resolution spin echo sequence: (**A**) *T*_1_ weighted and (**B**) *T*_2_ weighted.

Lastly, MRI in vitro experiments have been performed on a phantom composed of four vials containing suspensions of Gd-BAC-EDDS, Magnevist^®^ (*T*_1_ relaxing commercial CA), Endorem^®^ (*T*_2_ relaxing commercial CA) and ultrapure water are reported in Figure 9.

In particular, a standard high-resolution spin echo sequence was used to verify the MRI efficiency of Gd-BAC-EDDS both as a *T*_1_ and *T*_2_ relaxing agent.

It is apparent for both images that the ability of our system to contrast the image is in good agreement with the measured relaxivities at approximately 8 MHz, corresponding to the MRI tomograph operating field (0.2 T), a field region normally employed for musculoskeletal diagnosis in the clinic. Indeed, in a *T*_1_-weighted image (see Figure 9A) the signal of the Gd-BAC-EDDS suspension is brighter than the one of Magnevist and therefore shows better performance as a positive CA. On the other hand, in a *T*_2_-weighted image (see Figure 9B) the Gd-BAC-EDDS signal is darker than the one of Magnevist although it did not reach the level of the Endorem signal. Consequently, the efficacy of our material as a positive CA, and partially as a negative CA, was also confirmed by in vitro MRI experiments.

## 3. Conclusions

The PAA named BAC-EDDS, bearing several chelating moieties in each repeat unit, has been shown to tightly bind Gd^3+^ ions. The synthesis of BAC-EDDS took place with a sustainable process since it was synthesized at room temperature, in water at pH 10 and in the absence of added catalysts or organic solvents. It has been ascertained by DLS that the polymer coils aggregate in solution to form nanoparticles of ca. 150 nm hydrodynamic diameter, that increase in size up to 255 ± 90 nm when treated with 0.5 equivalents Gd^3+^ with respect to the monomer repeat units. Due to the inherent complexity of the BAC-EDDS structure, the protonation constants, obtained by adopting the De Levie method as well as Hyperquad fitting, were estimated not only in the polymer species but also in a purposely synthesized molecular model (Agly-EDDS), which mimics the structure of the BAC-EDDS repeating unit. Additionally, the stability constant for the 1:1 Gd-Agly-EDDS complex was estimated, resulting in a value (log K = 17.43) lying in the range of stability constants found in the literature for similar open polyaminopolycarboxylate ligands. The stoichiometry of the stable polymer complex was Gd_0_._5_-BAC-EDDS, so only the half of the repeating units were complexed and the other half remained in their free form. The NMRD curves for the longitudinal (*r*_1_) and transverse (*r*_2_) relaxivities were recorded over the frequency range 0.01–240 MHz to study the physical mechanisms of spin dynamics that accelerate the nuclear relaxation. The *r*_1_(ν) profiles have been successfully fitted with a model based on the Solomon–Bloembergen–Morgan theory. Among the other quantities obtained from the fitting procedure, the rotational correlation time *τ_r_* of 0.66 ns is particularly interesting and accounts for the Gd-BAC-EDDS molecular dimensions that differentiate it from the commercial CAs. In addition, in in vitro MRI experiments Gd-BAC-EDDS demonstrated the capacity to behave as an MRI contrast agent with higher performances in comparison to well-known commercial products. Therefore, the presented polymeric complex turned out to be a very interesting system that, in our opinion, would deserve further studies on its kinetic inertness and subsequent assessment of the in vivo performance as MRI contrast agent.

## 4. Materials and Methods

Materials and Instrumentations. All the chemicals were purchased from Sigma-Aldrich and used as received, if not otherwise specified. Ultrapure water (Milli-Q, Millipore, resistivity = 18 MΩ/cm^2^) was used for the preparation of the aqueous solutions. 2,2-Bis(acrylamido) acetic acid (BAC) was prepared by following a literature method [23] and purity was determined by NMR spectroscopy.

High-resolution NMR experiments were performed on a Bruker DRX400 spectrometer (Billerica, MA, USA) equipped with a Bruker 5 mm BBI Z-gradient probe head with a maximum gradient strength of 53.5 G/cm.

DLS and ζ-potential measurements were performed using a Malvern Zetasizer Nano ZS instrument at 25 °C and equipped with a 633 nm solid state He–Ne laser at a scattering angle of 173°, typically dissolving samples at 1 mg/mL concentration and averaging the measurements over at least three repeated runs.

UV−Vis absorption spectra were acquired on an Agilent model 8543 spectrophotometer (Santa Clara, CA, USA) at room temperature using standard 3 mL quartz cells with 1 cm path length.

ATR-FTIR spectra were acquired on a PerkinElmer Frontier instrument equipped with an ATR accessory with a diamond/ZnSe crystal (Waltham, MA, USA). The IR spectra were registered between 4000 and 400 cm^−1^.

Size exclusion chromatography (SEC) traces were obtained for all polymers with Toso-Haas TSK-gel G4000 PW and TSK-gel G3000 PW columns connected in series, using a Waters model 515 HPLC pump (Milano, Italy) equipped with a Knauer autosampler 3800 (Knauer, Bologna, Italy), a light scattering detector (670 nm), a viscometer Viscotek 270 dual detector (Malvern, Roma, Italy) and a refractive index detector (Model 2410, Waters, Milano, Italy). The mobile phase was a 0.1 M Tris buffer (pH 8.00 ± 0.05) solution with 0.2 M sodium chloride. Sample concentration: 20 mg mL^−1^; flow rate: 1 mL min^−1^; injection volume: 20 µL; loop size: 20 µL; column dimensions: 300 × 7.5 mm^2^. The instrument optical constants were determined using PEO 24 kDa as narrow standard. Before analysis, each sample was filtered through a 0.2 µm Whatman^TM^ syringe filter (Maidstone, UK).

pH measurements were performed using an AMEL 338 pH meter equipped with a Z113441-1EA glass microelectrode from Sigma-Aldrich.

Elemental C, H and N analyses were carried out on a PerkinElmer CHN 2400 instrument.

Mass spectrometry (ESI-MS) analysis was carried out on an ESI LCQ Fleet ion trap mass spectrometer (San Jose, CA USA) equipped with an HPLC UltiMate™ 3000, Thermo Fisher by dissolving Agly-EDDS in a 1:1 water/methanol mixture.

The ^1^H nuclear magnetic resonance (NMR) relaxometry profiles were collected at room temperature in the frequency range 10 kHz < ν < 240 MHz by measuring the longitudinal and the transverse nuclear relaxation times *T*_1_ and *T*_2_ of colloidal solutions of the different samples. The NMR signal detection and generation were obtained by a Smartracer Stelar relaxometer in the range 10 kHz < ν < 9.5 MHz, a Stelar Spinmaster and an Apollo-Tecmag Fourier Transform nuclear magnetic resonance (FT-NMR) spectrometer for ν > 9.5 MHz using the standard pulse sequences Saturation Recovery for *T*_1_ and Car–Purcell–Meiboom–Gill (CPMG) for *T*_2_.

In vitro MRI experiments were performed at room temperature on vials containing Gd-BAC-EDDS and commercial AC solutions at 8.5 MHz using an Artoscan Imager by Esaote SpA. The employed pulse sequence was a high-resolution spin echo sequence with TR/TE/NEX = 200 ms/18 ms/2, matrix = 256 × 256, FOV = 180 × 180 for the *T*_1_-weighted image, and with TR/TE/NEX = 5000 ms/80 ms/2, matrix = 256 × 192, FOV = 180 × 180 for the *T*_2_-weighted image. Here, TE is the echo time, TR the repetition time, NEX the number of averages and FOV the field of view. The suspension concentrations are Gd-BAC-EDDS = 0.87 mM, Magnevist = 0.83 mM, Endorem = 0.85 mM.

### 4.1. Synthesis of BAC-EDDS

In a single-necked flask (50 mL), fitted with a glass stopper and magnetic stirrer, LiOH (289.65 mg, 4.930 mmol) was dissolved in the EDDS aqueous solution (35% *w*/*w*, 3.5 mL, 4.930 mmol). Once the solution became clear, BAC (1 g, 4.930 mmol) and LiOH (289.65 mg, 4.930 mmol) were added. The reaction was left in the dark at RT for 5 days. At the end of this period, the solution was clear and viscous. The reaction mixture was taken up by adding some water to dilute the viscous mixture, acidified to pH 4.5 by the addition of aqueous 37% HCl, then precipitated by the addition of 60 mL acetone. To recover the precipitate, the mixture was centrifuged (5 min, 8000 rpm), the supernatant removed and a further washing with acetone was carried out. The precipitate polymer was dissolved in 30 mL Milli-Q water and ultrafiltered on Amicon with a 3000 Da cutoff membrane. The filtrate was finally lyophilized. ^1^H NMR [400 MHz, D_2_O]: δ_H_ 2.42 (4H, m), 2,68 (2H, m), 2.78 (2H, m), 3.14 (4H, m), 3.27 (2H, m), 3.45 (2H, m), 4.11 (2H, m), 5.46 (1H, s). ^13^C NMR [400 MHz, D_2_O]: δ_C_ 31.2, 33.3, 43.2, 47.6, 48.5, 57.4, 62.4.

### 4.2. Formation of the Polymer Complex by Fractioned Additions of Gd(III)

BAC-EDDS (40.0 mg) was dissolved in 1 mL Milli-Q water and added to 130 μL NaOH 1M to set the pH at 7.0. Contemporarily, a solution of GdCl_3_·6H_2_O (13.5 mg in 500 μL Milli-Q water) containing 0.5 equivalents Gd(III) with respect to the polymer repeat units was also prepared.was made. Then, the Gd(III) solution was fractionally added in 5 additions of 100 μL each. After each addition, the solution was vigorously shaken and the pH adjusted by adding further NaOH to bring its value back to ca. 7 to favor the re-dissolution of the possible forming precipitate. Finally, the solution was warmed at 70 °C for 1h. The purification from the possible Gd(III) fraction not tightly interacting with the polymer was achieved by the use of the Chelex100 ion exchange resin, which was added in the proper amounts to the polymeric complex solution and left to act for 1 h at room temperature. Then, the suspension was centrifuged and the supernatant solution lyophilized. The fluffy and white solid obtained was stored at 4 °C for further uses. Elemental analysis for C_18_H_24_N_4_O_12_GdCl_3_(NaCl)_2_._5_(H_2_O)_3_ cltd C, 26.37; H, 3.63; N, 6.83; found C, 25.86; H, 3.62; N, 6.60.

### 4.3. Xylenol Orange Colorimetric Test by UV–Vis Spectroscopy

A 4.26 mM stock solution of GdCl_3_·6H_2_O was prepared in Milli-Q water to be used for the calibration curve. Then, in a 50 mL volumetric flask, a second stock solution of Xylenol Orange in ammonium acetate buffer (pH 5.7) was prepared as follows: 364 μL of a 2.2 mM solution of Xylenol Orange in Milli-Q water were diluted in 40 mL Milli-Q water and then added to 0.14 mL of glacial acetic acid. The pH was set at 5.4 by slowly adding aqueous NH_3_ (28% *w*/*w*) and finally it was brought to volume by adding Milli-Q water. The so-prepared colorimetric indicator solution was then employed for the preparation of 4 solutions of concentrations 1, 2, 3, 4 μM whose UV–Vis spectrum was recorded and the calibration curve obtained by the absorbance ratio A_578 nm_/A_434 nm_. The solution of the Gd-BAC-EDDS polymeric complex was then added to the stock solution of Xylenol Orange and the free Gd(III) % mol/mol estimated was less than 0.2%.

### 4.4. Bulk Magnetic Susceptibility (BMS) Shift Measurements

After a solution of BAC-EDDS (40.4 mg in 1 mL Milli-Q water) was treated with 1 equivalent of GdCl_3_·6H_2_O (27.7 mg, 0.0718 mmol, dissolved in 300 μL Milli-Q water) with respect to the polymer repeating unit and made to interact as described in § 5.2, it was purified from the unbound Gd(III) by Chelex100 as described above, and only after the total Gd(III) was estimated by the BMS shift measurement. To this end, a coaxial NMR tube was employed, inserting in the inner part a D_2_O solution of 1% *v/v* tert-butanol and in the outer part 8.9 μL of the solution of Gd-BAC-EDDS and 3.0 μL of tert-butanol and diluted with D_2_O until 300 μL. The difference in the chemical shift in the methyl of tert-butanol in the outer and inner part (∆χ) was measured and, by applying Equation (2), the Gd(III) molar concentration was obtained, confirming that 54% Gd(III) was retained by BAC-EDDS.

### 4.5. ^17^O NMR-Induced Shift Measurements

BAC-EDDS (30 mg) was dissolved in 500 μL D_2_O directly in an NMR tube, then the pH was adjusted to 7.0 by the addition of 71 μL NaOH 1M. ^17^O NMR of the sample was recorded, and the sample was added to aliquots of 15 μL of a water solution of GdCl_3_·6H_2_O (20.5 mg in 100 μL water). After each addition, a ^17^O spectrum centered on the oxygen signal of water was acquired. The very same experiments were also conducted on a D_2_O sample by adding Gd(III) solution treated with the same amounts of Gd(III). The induced shift in the ^17^O water resonance was plotted for the two samples vs. Gd(III) μmols (see Figure S5).

### 4.6. Synthesis of Agly-EDDS Molecular Ligand

(a) First of all, acryloyl glycine was prepared by following a literature procedure [32]. Briefly, 10 g glycine (0.13 mol) were dissolved in 100 mL NaOH 3M and put in an ice bath to lower the temperature. Then, a 1,4-dioxane solution of acryloyl chloride (0.14 mol in 50 mL) was added drop by drop under vigorous stirring. The obtained solution was maintained under stirring for 1.5 h at 0 °C. Then, the mixture was washed with ethyl ether (3 × 50 mL). The aqueous fraction was acidified by adding HCl 4 M until pH 2 was reached, saturated with NaCl and extracted with ethyl acetate (5 × 40 mL). The organic fractions were collected and dried over MgSO_4_, the mixture filtered and the filtrate added with diethyl ether 1:1. The precipitate crystalline white solid was then put in a desiccator overnight. Yield 67.6%. Melting point 130.7–132 °C. ^1^H-NMR (D_2_O, 300 K, 9.4 T) δ 6.45–6.25 (m, 2H), 4.13 (s, 2H). (b) In a Schlenk tube under nitrogen and equipped with a magnetic stirrer, 200 mg acryloyl glycine (1.55 mmol) together with EDDS (7.8 mmol) were suspended in 1.6 mL Milli-Q water. After the addition of 150 μL NaOH 32% the suspension became completely clear. The mixture was heated at 50 °C in an oil bath, added to 100 μL NaOH 32% *w*/*w* and 1 mL water, and left for a total of 12 days under stirring and inert atmosphere, until the double bond signals were heavily reduced in the ^1^H NMR spectrum. To quench the reaction, the mixture was treated with a few microliters of HCl 30% until the pH dropped to ca. 1, while the solution remained clear. The purification was carried out by ethyl acetate extraction of the unreacted acryloyl glycine. The water fraction was then lyophilized, affording a white solid. ^1^H NMR (D_2_O, 300 K, 9.4 T) δ 4.28 (*H9*, dd, 1H), 3.95 (*H2*, s, 2H), 3.48–3.30 (*H8* and *H6*, m, 2H), 3.30–3.20 (*H8′* and *H6′*, m, 2H), 2.96 (*H11* and *H11′*, m, 2H). ^13^C NMR (D_2_O, 300 K, 9.4 T) δ 174.1 (*C1*), 173.3 (*C10* and *C12*), 172.0 (*C4*), 60.7 (*C9*), 49.0 (*C8*), 48.2 (*C6*), 41.1 (*C2*), 31.5 (*C11*), 31.1 (*C5*). Elemental analysis for C_20_H_30_N_4_O_14_(NaCl)_6_·(HCl)_2_(H_2_O) cltd C, 24.21; H, 3.45; N, 5.65; found C, 25.38; H, 3.57; N, 5.53. ESI-MS: *m*/*z* calcd: 550.18 [M]^+^, found [M + 1]^+^ 551.32; calcd: 573.17 [M-1 + Na]^+^, found 573.37 [M-1 + Na]^+^; calcd: 595.15 [M-2 + 2Na]^+^, found 595.34 [M-2 + 2Na]^+^; calcd: 617.13 [M-3 + 3Na]^+^, found 617.34 [M-3 + 3Na]^+^; calcd: 639.11 [M-4 + 4Na]^+^, found 639.33 [M-4 + 4Na]^+^.

### 4.7. Potentiometric Titrations

Equilibrium constants (protonation and metal complex stability constants) were determined by potentiometric titrations. To this end, we used an AMEL 338 pH-meter equipped with a Z113441-1EA glass microelectrode from Sigma-Aldrich. Prior to the titration, the electrode was calibrated by using 3 buffer solutions at pH 4.00, 7.00 and 9.00 (VWR, AVS Titrinorm). The ionic strength was set to 0.10 M by adding the proper amount of KCl. The titrations were carried out under nitrogen atmosphere in a thermostatted vessel at 25 °C, and equipped with a magnetic stirrer at a ligand concentration that varied in the range 2.3–6.0 × 10^−3^ M. The pH of the starting solution was adjusted at a value lower than 2 and the titration was carried out by subsequent additions of NaOH 0.1 M. Protonation constants were determined by simulating the experimental curves by the De Levie method in an Excel sheet (see Supplementary Materials and ref. [33]) as well as by fitting them with Hyperquad software, which was also used for the determination of the Gd-Agly-EDDS stability constants. For the stability constant determination, a solution of Agly-EDDS added to an equimolar amount of GdCl_3_ was titrated with NaOH 0.1 M using the same modality adopted for the protonation constant determination. The data treatment and processing were carried out with the program Hyperquad, taking into account all the possible simultaneous equilibria in solution including gadolinium hydroxide formation, protonation equilibria of both the complex and the free ligand and carbonate-based equilibria due to a small fraction of CO_2_ possibly dissolved in the solvent and in the titrant.

### 4.8. ^1^H NMR Titration on Agly-EDDS Ligand

A solution of Agly-EDDS (7.4 mg in 600 μL D_2_O) was prepared directly in a 5 mm NMR tube and the pH adjusted to ca. 1 with the addition of a few microliters of HCl. A series of ^1^H NMR spectra were acquired by suppressing the solvent signal after each addition of NaOH 0.1 M drops and after measuring the pD values directly in the NMR tube by a Z113441-1EA glass microelectrode (Darmstadt, Germany) from Sigma-Aldrich. The pD values were then converted to pH by the addition of 0.4 pH units, to account for the H/D isotopic effect [42].

## Figures and Tables

**Table 1:** Elemental analysis report for BAC-EDDS.

	Calculated for C_18_H_26_O_12_N_4_	Found	Calculated for C_18_H_26_O_12_N_4_ × 4H_2_O
C%	44.08	38.69	38.44
H%	5.34	6.17	6.09
N%	11.42	9.89	9.96
C/N	3.86	3.91	3.85

**Table 2:** Protonation constants for Agly-EDDS and BAC-EDDS (25 °C, 0.1 M KCl, N_2_).

	De Levie		Hyperquad	
	Agly-EDDS	BAC-EDDS	Agly-EDDS	BAC-EDDS
pK_a1_	ND	ND	ND	ND
pK_a2_	2.2 ± 0.1	ND	2.40 ± 0.12	ND
pK_a3_	3.0 ± 0.2	2.4 ± 0.1	3.00 ± 0.10	2.43 ± 0.03
pK_a4_	3.1 ± 0.2	3.6 ± 0.2	3.40 ± 0.10	3.40 ± 0.02
pK_a5_	3.7 ± 0.2	4.7 ± 0.2	3.85 ± 0.08	4.21 ± 0.02
pK_a6_	4.3 ± 0.2	6.0 ± 0.3	4.52 ± 0.06	5.72 ± 0.02
pK_a7_	5.3 ± 0.3	10.4 ± 0.5	5.86 ± 0.04	9.31 ± 0.01
pK_a8_	9.7 ± 0.5	---	9.34 ± 0.02	

**Table 3:** Thermodynamic parameters for 1:1 Gd-Agly-EDDS complex.

	Gd-Agly-EDDS (1:1)	
Constants	Log K	Log β
$K_{GdL}=\frac{\left[GdL\right]}{\left[Gd^{3+}\right]\left[L\right]}$	17.43 ± 0.07	17.43 ± 0.07
$K_{GdLH}=\frac{\left[GdLH\right]}{\left[GdL\right]\left[H^{+}\right]}$	7.68 ± 0.11	25.12 ± 0.04
$K_{GdLH_{2}}=\frac{\left[GdLH_{2}\right]}{\left[GdLH\right]\left[H^{+}\right]}$	3.56 ± 0.09	28.69 ± 0.05

## Data Availability

The data presented in this study are available in the article and in the Supplementary Materials.

## Supplementary Materials

Figure S1: FTIR-Spectrum of BAC-EDDS. Figure S2: UV-vis absorption spectrum of BAC-EDDS. Figure S3: DLS and ζ-potential measurements of a sample of Gd_0_._5_BAC-EDDS. Figure S4: UV-vis absorption spectra of a solution of Xylenol Orange at increasing amounts of added Gd(III) salt at buffered pH. Figure S5: ^1^H NMR of *tert*-butanol solution after two successive additions of the polymeric complex in the outer part of the coaxial tube for the measurement of the chemical shift difference (∆χ) of the methyl groups of *tert*-butanol in the two compartments of the coaxial NMR tube. Figure S6: ^17^O NMR shift of water vs. Gd(III) μmols added to a solution of [Gd(H_2_O)_9_]^3+^ and BAC-EDDS-Gd. Figure S7: ^1^H-^1^H COSY NMR experiment of Agly-EDDS in D_2_O. Figure S8: ^1^H-^1^H NOESY NMR experiment of Agly-EDDS in D_2_O. Figure S9: ^1^H-^13^C HSQC-DEPT NMR experimento of Agly-EDDS in D_2_O. Figure S10: ESI-MS spectrum of a sample of Agly-EDDS at acidic pH (H_2_O). Figure S11: Prevailing intervals for Agly-EDDS and BAC-EDDS. Figure S12: ^1^H NMR titration of Agly-EDDS. De Levie treatment for Agly-EDDS monomer model as well as for BAC-EDDS are reported.

## Sample Availability

Samples of the compounds BAC-EDDS and Agly-EDDS are available from the authors.
