# Supplementary material for: Nanosized T1 MRI Contrast Agent Based on a Polyamidoamine as Multidentate Gd Ligand
Source: Molecules. 2021 Dec 28;27(1):174. doi: 10.3390/molecules27010174 (PMC8746954; doi:10.3390/molecules27010174)
Supplement: Supplementary file 1 [file molecules-27-00174-s001.zip › molecules-1447064(1)-done.pdf]

Supplementary Materials

Nanosized T_1 MRI Contrast Agent Based on a Polyamidoamine as Multidentate Gd Ligand

Paolo Arosio [1,2,*], Davide Cicolari [3], Amedea Manfredi [4], Francesco Orsini [1,2], Alessandro Lascialfari [3], Elisabetta Ranucci [4], Paolo Ferruti [4] and Daniela Maggioni [2,4,*]

[1] Dipartimento di Fisica, INFN, Istituto Nazionale di Fisica Nucleare-Milano Unit, Università degli Studi di Milano, Via Celoria 16, 20133 Milano, Italy
[2] Consorzio Interuniversitario Nazionale per la Scienza e Tecnologia dei Materiali, Via Golgi 19, 20133 Milano, Italy
[3] Dipartimento di Fisica and INFN, Università degli Studi di Pavia, Via Bassi 6, 27100 Pavia, Italy
[4] Dipartimento di Chimica, Università degli Studi di Milano, Via Golgi 19, 20133 Milano, Italy

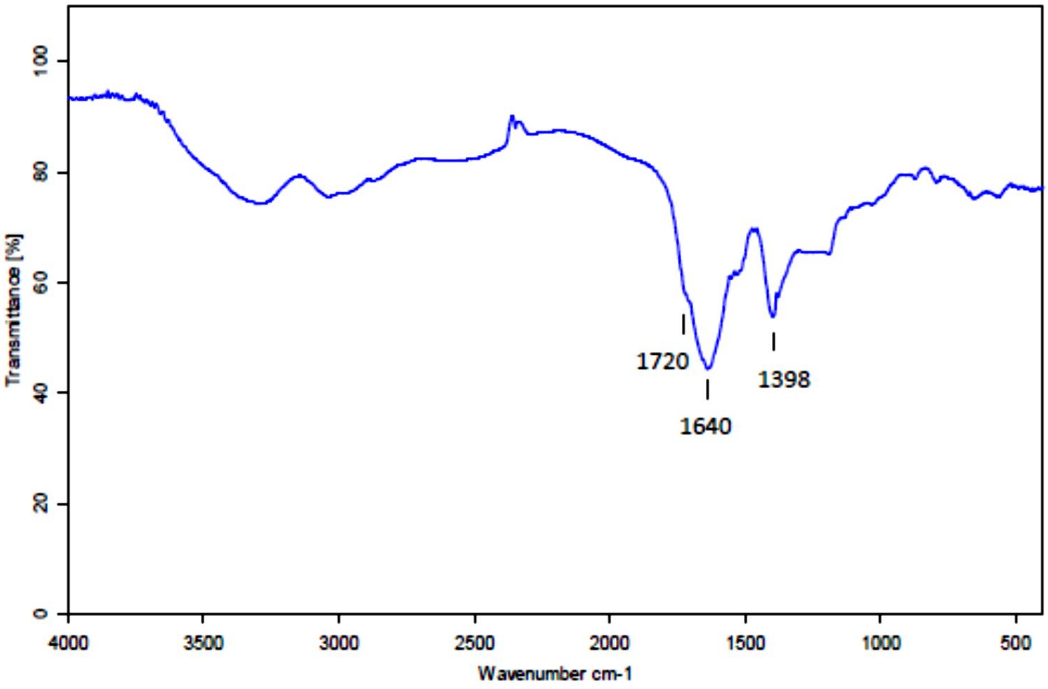

Figure S1. FTIR-Spectrum of BAC-EDDS, which was obtained by depositing an aqueous solution (few drops) on a pre-formed KBr pellet and gently warming it to remove water by evaporation.

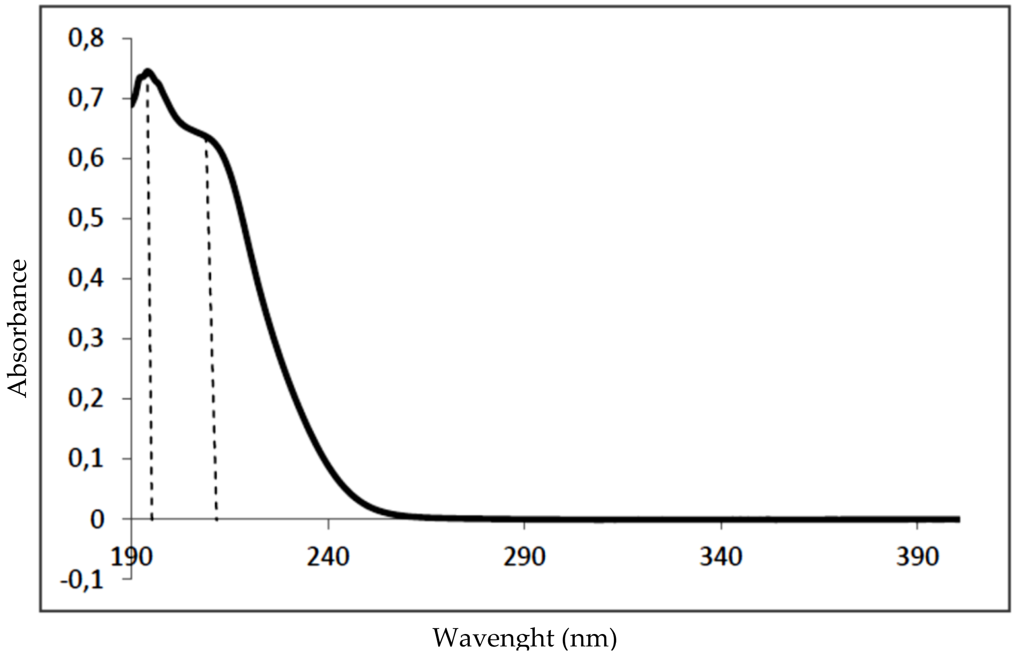

Figure S2. UV-vis absorption spectrum of homopolymer BAC-EDDS in water (5 μL of 0.036 M BAC-EDDS diluted in 700 μL milliQ water).

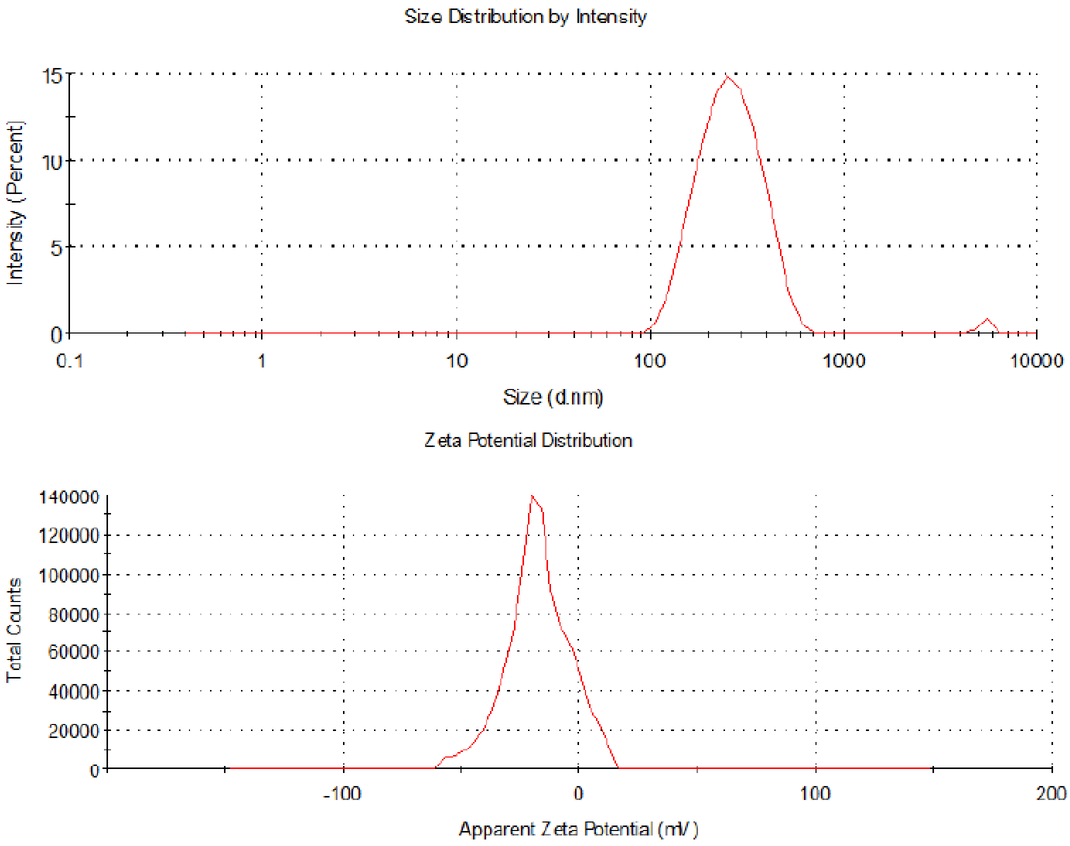

Figure S3. DLS (by intensities, top) and ζ-potential measurements of a sample of $Gd_{0.5}$BAC-EDDS at pH ca 7 (milliQ water, 25 °C).

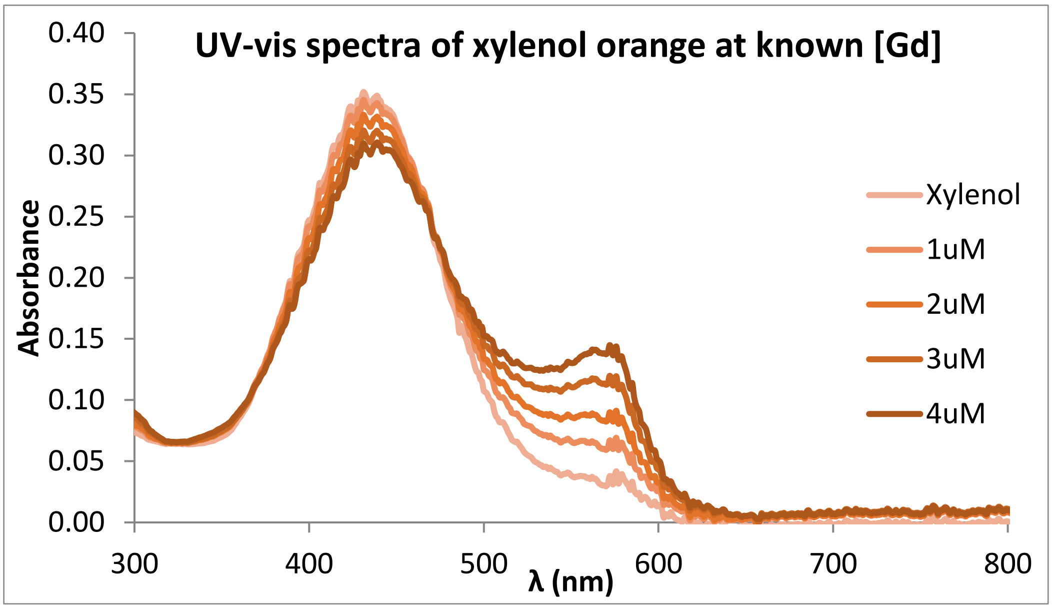

Figure S4. UV-vis absorption spectra of a solution of Xylenol Orange at increasing amounts of added Gd(III) salt at buffered pH.

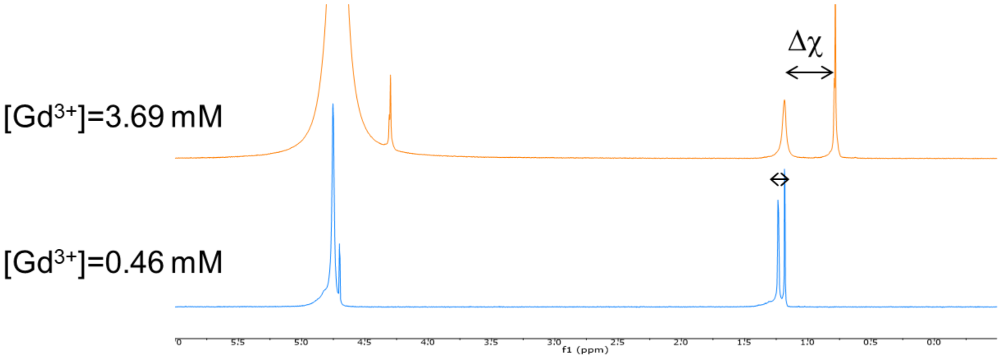

	Δχ	$[Gd^{3+}]_{tot}$ measured	$[Gd^{3+}]_{max}$	% of Gd retained by BAC-EDDS
**1st add**	0.0510	0.46 mM	0.9 mM	51.1%
**2nd add**	0.4021	3.69 mM	7.1 mM	51.9%

Figure S5. ^{1}H NMR of *tert*-butanol solution after two successive additions of the polymeric complex in the outer part of the coaxial tube for the measurement of the chemical shift difference (Δχ) of the methyl groups of *tert*-butanol in the two compartments of the coaxial NMR tube In the table below there are resumed the results of the analysis.

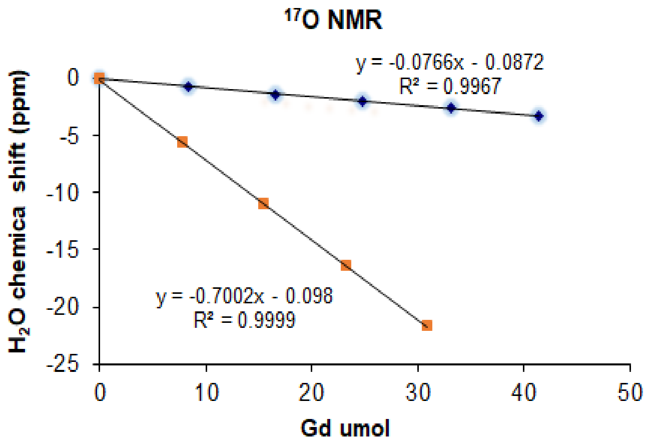

Figure S6. ^{17}O NMR shift of water vs. Gd(III) μmols added to a solution of $[Gd(H_2O)_9]^{3+}$ (orange squares) and BAC-EDDS-Gd (blue diamonds).

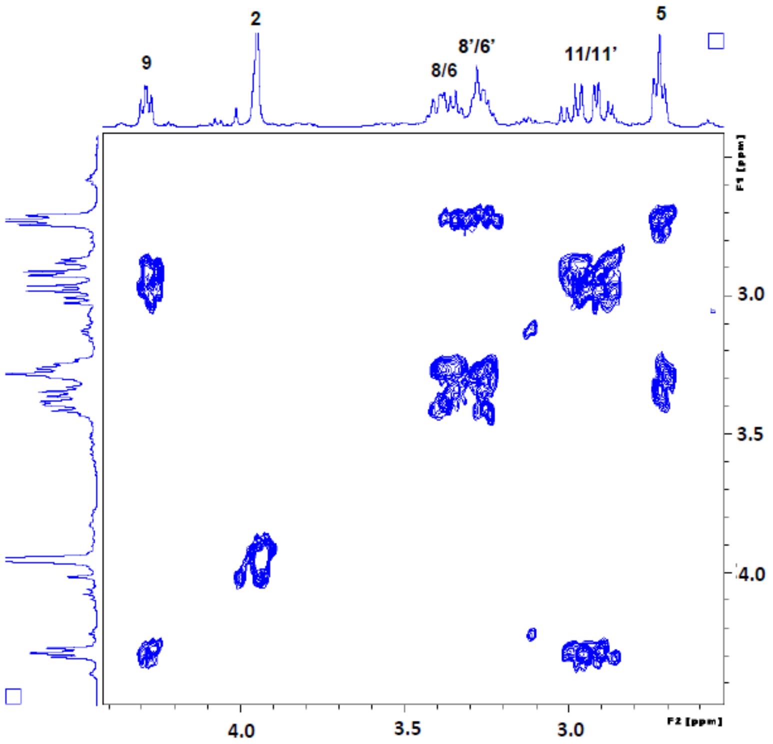

Figure S7. ^1H-^1H COSY of Agly-EDDS at acid pH (D_2O, 300 K,9.4 T).

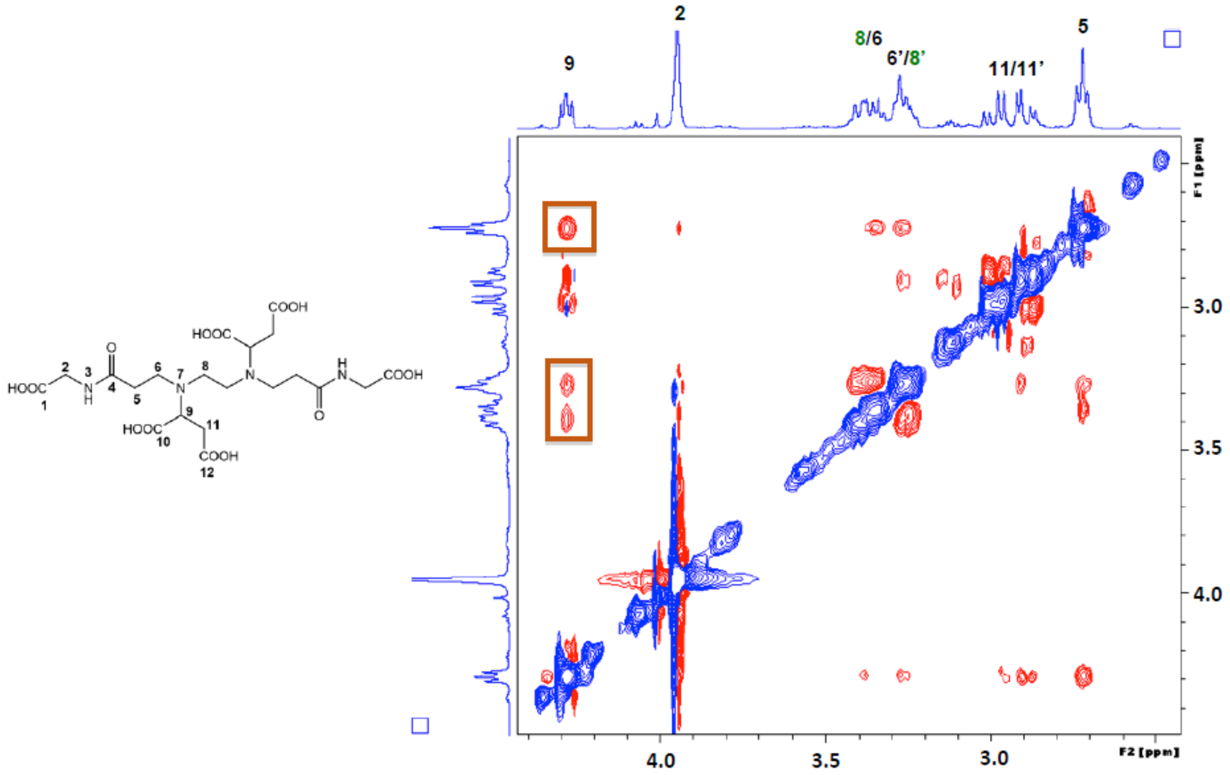

Figure S8. ^{1}H-^{1}H NOESY experiment of a Agly-EDDS sample at acidic pH (D_2O, 300 K, 9.4 T).

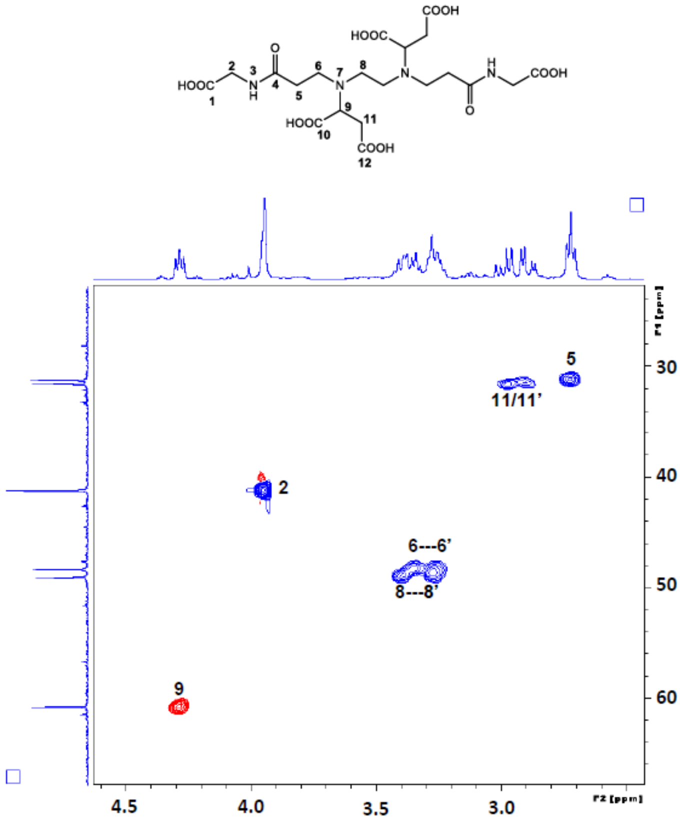

Figure S9. ^{1}H-^{13}C HSQC of a sample of Agly-EDDS at acidic pH (D_2O, 300 K, 9.4 T).

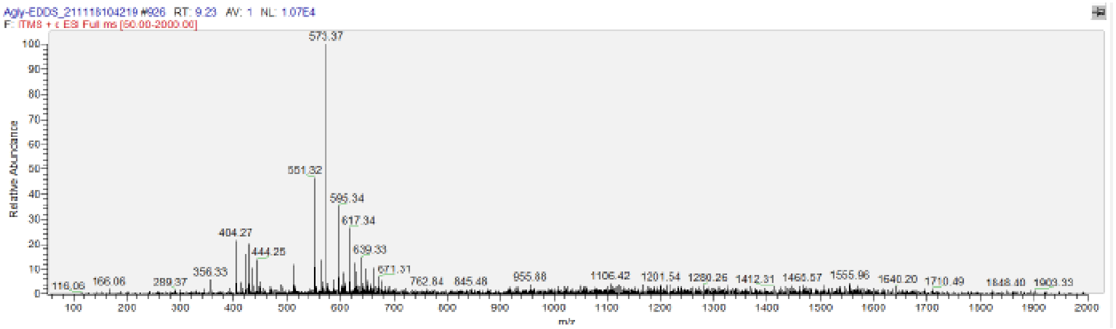

Figure S10. ESI-MS spectrum of a sample of Agly-EDDS at acidic pH (H2O).

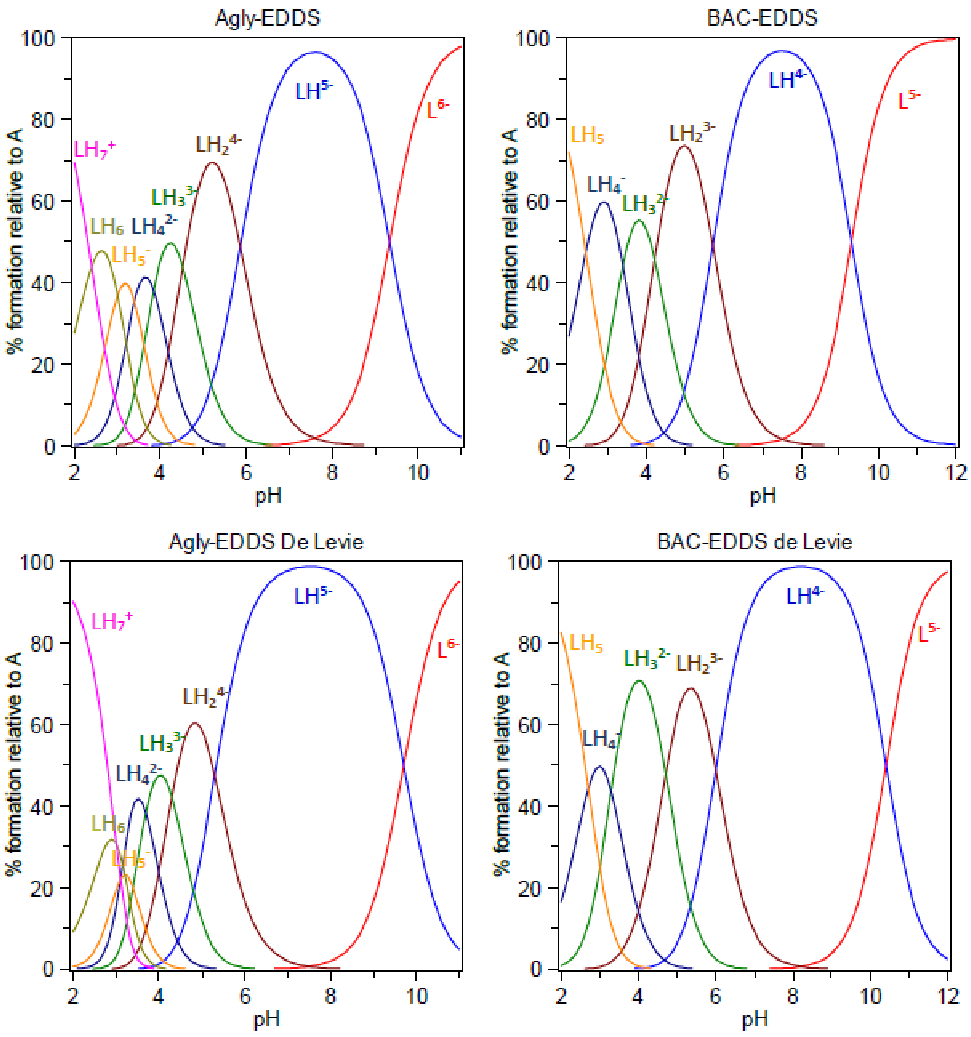

Figure S11. Prevailing intervals for Agly-EDDS and BAC-EDDS, respectively, plotted on the bases of the estimated pKa values with Hyperquad (top) and De Levie simulation (bottom).

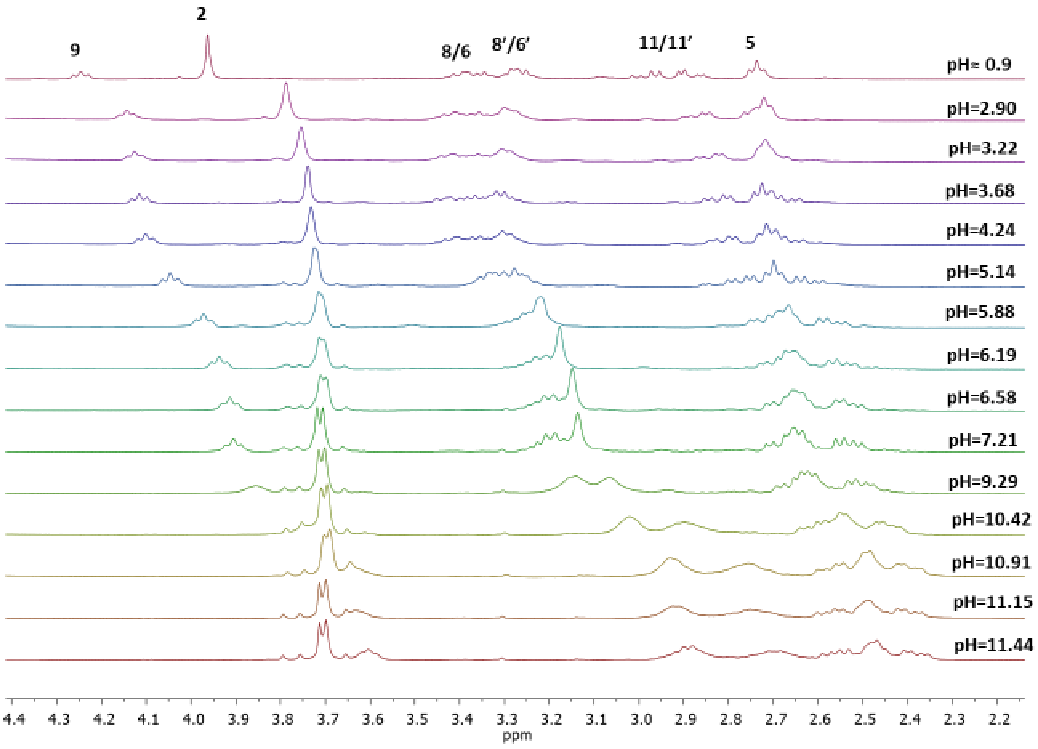

Figure S12. 1H NMR titration of Agly-EDDS (D_2O, 9.4 T, 300 K).

De Levie treatment for Agly-EDDS monomer model

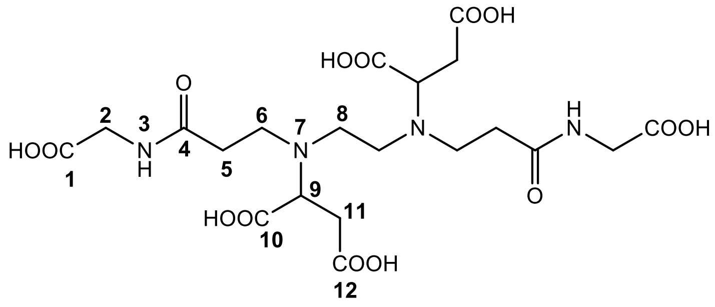

From now on Agly-EDDS in its neutral form will be indicated as LH_6.

The dissociation function F_a, defined as the sum of all the fraction of species α_i of the charged species deriving from LH_6 for protonation or deprotonation

$$F_a = -2\alpha_0 - \alpha_1 + \alpha_3 + 2\alpha_4 + 3\alpha_5 + 4\alpha_6 + 5\alpha_7 + 6\alpha_8$$

Where the species in equilibrium in solution are LH_8^{2+}, LH_7^{+}, LH_6, LH_5^{-}, LH_4^{2-}, LH_3^{3-}, LH_2^{4-}, LH^{5-}, L^{6-}.

The relative dissociation constants K_a as a function of the activity of the various species are expressed as follows:

$$k_{a1} = \frac{[LH_7^+]\cdot[H^+]}{[LH_8^{2+}]\cdot\gamma^2}$$

$$k_{a2} = \frac{[LH_6]\cdot[H^+]}{[LH_7^+]}$$

$$k_{a3} = \frac{[LH_5^-]\cdot[H^+]\cdot\gamma^2}{[LH_6]}$$

$$k_{a4} = \frac{[LH_4^{2-}] \cdot [H^+] \cdot \gamma^4}{[LH_5^-]}$$

$$k_{a5} = \frac{[LH_3^{3-}] \cdot [H^+] \cdot \gamma^6}{[LH_4^{2-}]}$$

$$k_{a6} = \frac{[LH_2^{4-}] \cdot [H^+] \cdot \gamma^8}{[LH_3^{3-}]}$$

$$k_{a7} = \frac{[LH^{5-}] \cdot [H^+] \cdot \gamma^{10}}{[LH_2^{4-}]}$$

$$k_{a8} = \frac{[L^{6-}] \cdot [H^+] \cdot \gamma^{12}}{[LH^{5-}]}$$

where $\gamma \cong \gamma^{(z)_i^2} = 10^{\left[-0.5\left(\frac{\sqrt{\mu}}{1+\sqrt{\mu}}-0.3\mu\right)\right]}(\mu = ionic\ stregth, Davies\ equation.$

At this point, we want to obtain the concentration expressions as a function of the species L^{6-} concentration, so by successive substitutions we obtain the following equations:

$$[LH_8^{2+}] = \frac{[L^{6-}] * [H^+]^8 * [\gamma]^{40}}{k_{a1} * k_{a2} * k_{a3} * k_{a4} * k_{a5} * k_{a6} * k_{a7} * k_{a8}}$$

$$[LH_7^+] = \frac{[L^{6-}] * [H^+]^7 * [\gamma]^{42}}{k_{a2} * k_{a3} * k_{a4} * k_{a5} * k_{a6} * k_{a7} * k_{a8}}$$

$$[LH_6] = \frac{[L^{6-}] * [H^+]^6 * [\gamma]^{42}}{k_{a3} * k_{a4} * k_{a5} * k_{a6} * k_{a7} * k_{a8}}$$

$$[LH_5^-] = \frac{[L^{6-}] * [H^+]^5 * [\gamma]^{40}}{k_{a4} * k_{a5} * k_{a6} * k_{a7} * k_{a8}}$$

$$[LH_4^{2-}] = \frac{[L^{6-}] * [H^+]^4 * [\gamma]^{36}}{k_{a5} * k_{a6} * k_{a7} * k_{a8}}$$

$$[LH_3^{3-}] = \frac{[L^{6-}] * [H^+]^3 * [\gamma]^{3}}{k_{a6} * k_{a7} * k_{a8}}$$

$$[LH_2^{4-}] = \frac{[L^{6-}] * [H^+]^2 * [\gamma]^{22}}{k_{a7} * k_{a8}}$$

$$[LH^{5-}] = \frac{[L^{6-}] * [H^+] * [\gamma]^{12}}{k_{a8}}$$

The concentration of Agly-EDDS in each instant is described as follows:

$$[Agly - EDDS] = [L^{6-}] \cdot D$$

where D is expressed by the following equation:

$$D \equiv \left(\frac{[H^+]^8 \cdot [\gamma]^{40}}{k_{a1} \cdot k_{a2} \cdot k_{a3} \cdot k_{a4} \cdot k_{a5} \cdot k_{a6} \cdot k_{a7} \cdot k_{a8}} + \frac{[H^+]^7 \cdot [\gamma]^{42}}{k_{a2} \cdot k_{a3} \cdot k_{a4} \cdot k_{a5} \cdot k_{a6} \cdot k_{a7} \cdot k_{a8}} + \frac{[H^+]^6 \cdot [\gamma]^{42}}{k_{a3} \cdot k_{a4} \cdot k_{a5} \cdot k_{a6} \cdot k_{a7} \cdot k_{a8}} + \frac{[H^+]^5 * [\gamma]^{40}}{k_{a4} \cdot k_{a5} \cdot k_{a6} \cdot k_{a7} \cdot k_{a8}} + \frac{[H^+]^4 * [\gamma]^{36}}{k_{a5} \cdot k_{a6} \cdot k_{a7} \cdot k_{a8}} + \frac{[H^+]^3 \cdot [\gamma]^{3}}{k_{a6} \cdot k_{a7} \cdot k_{a8}} + \frac{[H^+]^2 \cdot [\gamma]^{22}}{k_{a7} \cdot k_{a8}} + \frac{[H^+] \cdot [\gamma]^{12}}{k_{a8}} + 1\right)$$

while the fraction of species α can be written as follows, where [Agly-EDDS] is the analytical concentration of Agly-EDDS:

$$\alpha_0 = \frac{[LH_8^{2+}]}{[Agly - EDDS]} = \frac{[H^+]^8 \cdot [\gamma]^{40}}{D}$$

$$\alpha_1 = \frac{[LH_7^+]}{[Agly - EDDS]} = \frac{k_1 * [H^+]^7 \cdot [\gamma]^{42}}{D}$$

$$\alpha_2 = \frac{[LH_6]}{[Agly - EDDS]} = \frac{k_1 \cdot k_2[H^+]^6 \cdot [\gamma]^{42}}{D}$$

$$\alpha_3 = \frac{[LH_5^-]}{[Agly - EDDS]} = \frac{k_1 \cdot k_2 \cdot k_3 \cdot [H^+]^5 \cdot [\gamma]^{40}}{D}$$

$$\alpha_4 = \frac{[LH_4^{2-}]}{[Agly - EDDS]} = \frac{k_1 \cdot k_2 \cdot k_3 \cdot k_4 \cdot [H^+]^4 \cdot [\gamma]^{36}}{D}$$

$$\alpha_5 = \frac{[LH_3^{3-}]}{[Agly - EDDS]} = \frac{k_1 \cdot k_2 \cdot k_3 \cdot k_4 \cdot k_5 \cdot [H^+]^3 \cdot [\gamma]^{30}}{D}$$

$$\alpha_6 = \frac{[LH_2^{4-}]}{[Agly - EDDS]} = \frac{k_1 \cdot k_2 \cdot k_3 \cdot k_4 \cdot k_5 \cdot k_6 \cdot [H^+]^2 \cdot [\gamma]^{22}}{D}$$

$$\alpha_7 = \frac{[LH^{5-}]}{[Agly - EDDS]} = \frac{k_1 \cdot k_2 \cdot k_3 \cdot k_4 \cdot k_5 \cdot k_6 \cdot k_7 \cdot [H^+] \cdot [\gamma]^{12}}{D}$$

$$\alpha_8 = \frac{[L^{6-}]}{[Agly - EDDS]} = \frac{k_1 \cdot k_2 \cdot k_3 \cdot k_4 \cdot k_5 \cdot k_6 \cdot k_7 \cdot k_8}{D}$$

Finally, the charge balance in solution is represented by the following equation, where NaCl appears since present in the lyophilized Agly-EDDS and HCl is added to fix the starting pH value:

$$[H^+] + [Na^+] + 2[LH_8^{2+}] + [LH_7^+] = [OH^-] + [Cl^-] + [LH_5^-] + 2[LH_4^{2-}] + 3[LH_3^{3-}] + 4[LH_2^{4-}] + 5[LH^{5-}] + 6[L^{6-}]$$

while the ionic strength is as follows:

$$\mu = \frac{1}{2} * ([H^+] + [OH^-] + [Na^+] + [Cl^-] + 2^2[LH_8^{2+}] + [LH_7^+] + [LH_5^-] + 2^2[LH_4^{2-}] + 3^2[LH_3^{3-}] + 4^2[LH_2^{4-}] + 5^2[LH^{5-}] + 6^2[L^{6-}])$$

In the μ calculation, the number of Na^+ ions are due to different contributions: the Na^+ that each Agly-EDDS brings (a number between 4 and 5, estimated by elemental analysis), the employed titrant NaOH, the support electrolyte (NaCl) added to maintain constant the ionic strength:

$$[Na^+] = 4.4[Agly - EDDS] + V_{NaOH} \cdot M_{NaOH} + [NaCl]$$

$$[Cl^-] = [NaCl] + [HCl]$$

Now, substituting in the first equation of F_a the expressions of α_i, we obtain the equation for the titrating volume V_b:

$$V_{NaOH} = \frac{N + \{([Agly - EDDS] \cdot (G - 2\alpha_0 - \alpha_1 + \alpha_3 + 2\alpha_4 + 3\alpha_5 + 4\alpha_6 + 5\alpha_7 + 6\alpha_8) - \Delta) \cdot V_{Agly-EDDS}\}}{[NaOH] + \Delta}$$

where N is the number of strong acid moles which have been added to the solution at the beginning of the titration, while G are the moles of Na^+ dissolved together with the ligand.

De Levie treatment for BAC-EDDS polyamidoamine

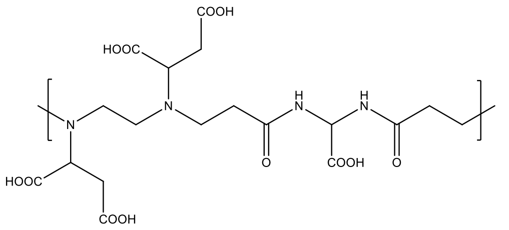

Unlike Agly-EDDS, BAC-EDDS has a total of 7 protonation sites, therefore the species in equilibrium in solution are: LH_7^{2+}, LH_6^{+}, LH_5, LH_4^{-}, LH_3^{2-}, LH_2^{3-}, LH^{4-}, L^{5-}. Proceeding as we did in the case of Agly-EDDS, we express the dissociation constants k_a as follows:

$$k_{a1} = \frac{[LH_6^+] * [H^+]}{[LH_7^{2+}] * \gamma^2}$$

$$k_{a2} = \frac{[LH_5] * [H^+]}{[LH_6^+]}$$

$$k_{a3} = \frac{[LH_4^-] * [H^+] * \gamma^2}{[LH_5]}$$

$$k_{a4} = \frac{[LH_3^{2-}] * [H^+] * \gamma^4}{[LH_4^-]}$$

$$k_{a5} = \frac{[LH_2^{3-}] * [H^+] * \gamma^6}{[LH_3^{2-}]}$$

$$k_{a6} = \frac{[LH^{4-}] * [H^+] * \gamma^8}{[LH_2^{3-}]}$$

$$k_{a7} = \frac{[L^{5-}] * [H^+] * \gamma^{10}}{[LH^{4-}]}$$

Then, we derive the concentration of the species as a function of L^{5-}:

$$[LH_7^{2+}] = \frac{[L^{5-}] * [H^+]^7 * [\gamma]^{28}}{k_{a1} * k_{a2} * k_{a3} * k_{a4} * k_{a5} * k_{a6} * k_{a7}}$$

$$[LH_6^+] = \frac{[L^{5-}] * [H^+]^6 * [\gamma]^{30}}{k_{a2} * k_{a3} * k_{a4} * k_{a5} * k_{a6} * k_{a7}}$$

$$[LH_5] = \frac{[L^{5-}] * [H^+]^5 * [\gamma]^{30}}{k_{a3} * k_{a4} * k_{a5} * k_{a6} * k_{a7}}$$

$$[LH_4^-] = \frac{[L^{5-}] * [H^+]^4 * [\gamma]^{28}}{k_{a4} * k_{a5} * k_{a6} * k_{a7}}$$

$$[LH_3^{2-}] = \frac{[L^{5-}] * [H^+]^3 * [\gamma]^{24}}{k_{a5} * k_{a6} * k_{a7}}$$

$$[LH_2^{3-}] = \frac{[L^{5-}] * [H^+]^2 * [\gamma]^{18}}{k_{a6} * k_{a7}}$$

$$[LH^{4-}] = \frac{[L^{5-}] * [H^+] * [\gamma]^{10}}{k_{a7}}$$

The concentration of BAC-EDDS is equal to:

$$[BAC - EDDS] = [L^{5-}] * D$$

where

$$D \equiv \left(\frac{[L^{5-}]*[H^+]^7*[\gamma]^{28}}{k_{a1}*k_{a2}*k_{a3}*k_{a4}*k_{a5}*k_{a6}*k_{a7}}+\frac{[L^{5-}]*[H^+]^6*[\gamma]^{30}}{k_{a2}*k_{a3}*k_{a4}*k_{a5}*k_{a6}*k_{a7}}+\frac{[L^{5-}]*[H^+]^5*[\gamma]^{30}}{k_{a3}*k_{a4}*k_{a5}*k_{a6}*k_{a7}} +\frac{[L^{5-}]*[H^+]^4*[\gamma]^{28}}{k_{a4}*k_{a5}*k_{a6}*k_{a7}}+\frac{[L^{5-}]*[H^+]^3*[\gamma]^{24}}{k_{a5}*k_{a6}*k_{a7}}+\frac{[L^{5-}]*[H^+]^2*[\gamma]^{18}}{k_{a6}*k_{a7}}+\frac{[L^{5-}]*[H^+]*[\gamma]^{10}}{k_{a7}}+1\right)$$

Consequently, the dissociation degrees α are as follows:

$$\alpha_0 = \frac{[LH_7^{2+}]}{[EDDS-BAC]} = \frac{[H^+]^7*[\gamma]^{28}}{D}$$

$$\alpha_1 = \frac{[LH_6^{+}]}{[EDDS-BAC]} = \frac{k_1*[H^+]^6*[\gamma]^{30}}{D}$$

$$\alpha_2 = \frac{[LH_5]}{[EDDS-BAC]} = \frac{k_1*k_2[H^+]^5*[\gamma]^{30}}{D}$$

$$\alpha_3 = \frac{[LH_4^{-}]}{[EDDS-BAC]} = \frac{k_1*k_2*k_3*[H^+]^4*[\gamma]^{28}}{D}$$

$$\alpha_4 = \frac{[LH_3^{2-}]}{[EDDS-BAC]} = \frac{k_1*k_2*k_3*k_4*[H^+]^3*[\gamma]^{24}}{D}$$

$$\alpha_5 = \frac{[LH_2^{3-}]}{[EDDS-BAC]} = \frac{k_1*k_2*k_3*k_4*k_5*[H^+]^2*[\gamma]^{18}}{D}$$

$$\alpha_6 = \frac{[LH^{4-}]}{[EDDS-BAC]} = \frac{k_1*k_2*k_3*k_4*k_5*k_6*[H^+]*[\gamma]^{10}}{D}$$

$$\alpha_7 = \frac{[L^{5-}]}{[EDDS-BAC]} = \frac{k_1*k_2*k_3*k_4*k_5*k_6*k_7}{D}$$

For the study of the pK_a of the polymer we took into account the possible carbonates dissolved in the solution for better fitting the titration curve at high pH values. The CO_2 concentration is given by the following equation:

$$[CO_2] = \frac{(\beta_0+\beta_1+\beta_2)*V_{EDDS-BAC}*[CO_2]_0}{V_{EDDS-BAC}+V_{NaOH}}$$

where:

$$[CO_2]_0 = fraction\ of\ [CO_2]*[BACEDDS]$$

is the analytical concentration of CO_2 in water, while the β parameters are the dissociation degrees relative to the species that CO_2 assumes in aqueous solution (CO_2, HCO_3^- and CO_3^{2-}):

$$\beta_0 = \frac{[H^+]^2}{D}$$

$$\beta_1 = \frac{[H^+]*K_{a1}}{D}$$

$$\beta_2 = \frac{K_{a1}*K_{a2}}{D}$$

where K_{a1} and K_{a2} are the dissociation constants for H_2CO_3 (pK_{a1}= 6.3; pK_{a2} = 10.3) and D is equal to

$$D \equiv [H^+]^2+[H^+]*K_{a1}+K_{a1}*K_{a2}$$

The ionic strength is calculated taking into account the employed tetrasodic form, the titrant used (NaOH), the supporting electrolyte (NaCl), and the HCl added at the beginning of the titration to lower the pH value:

$$\mu = \frac{1}{2}*([H^+]+[OH^-]+[Na^+]+[Cl^-]+2^2[LH_7^{2+}]+[LH_6^{+}]+[LH_4^{-}]+2^2[LH_3^{2-}]+3^2[LH_2^{3-}]+4^2[LH^{4-}]+5^2[LH^{5-}])$$

$$[Na^+] = 4[EDDS - BAC] + V_{NaOH} * M_{NaOH} + [NaCl]$$

$$[Cl^-] = [NaCl] + [HCl]$$

Lastly, the charge balance needs to be taken into account:

$$[H^+] + [Na^+] + 2[LH_7^{2+}] + [LH_6^+] = [OH^-] + [Cl^-] + [HCO_3^-] + 2[CO_3^{2-}] + [LH_4^-] + 2[LH_3^{2-}] + 3[LH_2^{3-}] + 4[LH^{4-}] + 5[L^{5-}]$$

Finally, as done for Agly-EDDS, the equation of the titrating volume is obtained:

$$V_{NaOH} = \frac{N + V_{EDDS-BAC} * \{[EDDS - BAC] * (G - 2\alpha_0 - \alpha_1 + \alpha_3 + 2\alpha_4 + 3\alpha_5 + 4\alpha_6 + 5\alpha_7) + [CO_2]_0 * (\beta_1 + 2\beta_2) - \Delta\}}{[NaOH] + \Delta}$$

where N are the added HCl moles that both lowered the starting pH and re-protonated the polymer, while G indicates the Na^+ moles which have been brought into solution per moles of ligand.
